# Multi-Platform Transcriptomic Classification of LUAD Driver Mutation Subtypes via Heterogeneous Ensemble Learning

**DOI:** 10.3390/diagnostics16203413

**Published:** 2026-10-20

**Authors:** Ilkay Sibel Kervancı, Gözde Özsert Yiğit

**Affiliations:** Computer Engineering Department, Gaziantep University, University Boulevard, Şehitkamil, 27310 Gaziantep, Turkey; skervanci@gantep.edu.tr

**Keywords:** LUAD, driver mutation classification, ensemble learning, Bayesian hyperparameter optimization, SHAP explainability, multi-cohort batch harmonization

## Abstract

**Background:** Accurate identification of EGFR-mutant, KRAS-mutant, and triple-negative (TN) LUAD driver mutation subtypes is essential for guiding targeted therapy, yet standard molecular testing is invasive and not always feasible. Gene expression-based machine learning offers an alternative that avoids additional invasive sampling; however, existing models are largely restricted to binary classification and lack cross-platform generalizability. **Methods:** We developed a multi-cohort, ComBat-harmonized pipeline integrating SHAP-driven feature selection and a Bayesian-optimized heterogeneous ensemble of five learners, extended with NSGA-II Pareto-optimal weight optimization under leak-free cross-validation. External validation used an independent RNA-seq cohort with reference-batch harmonization and threshold calibration. **Results:** ParetoVoting achieved the best internal performance (F1-macro = 0.736, AUROC = 0.877), statistically indistinguishable from SoftVoting (F1-macro = 0.735; Cliff’s δ=−0.12), which was carried forward for external validation. On TCGA-LUAD (n=508), SoftVoting attained F1-macro = 0.700 and AUROC = 0.850, though this reflects an out-of-range KRAS threshold (representative value ∼0.66–0.68). Ablation analysis showed components contributed unequally within internal cross-validation, with ComBat’s critical role instead emerging during cross-platform external generalization. SHAP analysis identified biologically coherent signatures consistent with known LUAD oncogenic signaling. **Conclusions:** This framework provides a reproducible, cross-platform approach to three-class driver mutation subtyping from transcriptomic data, offering benchmarks for this underexplored task and potential clinical utility where tissue-based profiling is infeasible.

## 1. Introduction

Lung cancer is the leading cause of cancer-related deaths worldwide, with approximately 1.8 million people dying each year according to 2022 GLOBOCAN data [1]. Non-small-cell lung cancer (NSCLC) accounts for approximately 85% of all lung cancer cases, with lung adenocarcinoma (LUAD) constituting the majority of these. With advancements in personalized medicine and precision oncology, identifying specific driver mutations has become critical for classifying NSCLC patients based on their treatment response. Currently, molecular alterations in driver genes such as Epidermal Growth Factor Receptor (EGFR), Kirsten Rat Sarcoma Viral Oncogene Homolog (KRAS), Anaplastic Lymphoma Kinase (ALK), ROS Proto-Oncogene 1 Receptor Tyrosine Kinase (ROS1), B-Raf Proto-Oncogene, Serine/Threonine Kinase (BRAF), Human Epidermal Growth Factor Receptor 2 (HER2), Mesenchymal–Epithelial Transition Factor (Hepatocyte Growth Factor Receptor) (MET), and Rearranged during Transfection (RET) serve as the primary guide in determining treatment strategies.

EGFR mutations are observed in 30–50% of Asian populations and 15–20% of Western populations in LUAD cases; in these patients, significant prolongation of overall survival is achieved with first-line treatment using tyrosine kinase inhibitors (TKIs) [2]. KRAS mutations, on the other hand, are the most common driver oncogenes in NSCLC, occurring at a rate of 25–35%, and were long considered “untargetable”; however, this view has begun to shift with the introduction of KRAS G12C inhibitors such as sotorasib and adagrasib into clinical practice [3,4]. The TN subtype—cases lacking any known driver mutations—is associated with a poor prognosis due to insufficient benefit from targeted therapies and constitutes the most challenging group of NSCLC to manage [5,6]. Therefore, the accurate differentiation of these three subtypes has become an indispensable requirement for making patient-specific treatment decisions.

Currently, tumor DNA sequencing and immunohistochemistry performed via tissue biopsy are considered the gold standard for identifying driver mutations in NSCLC. However, these methods have significant limitations, including the need for invasive biopsy, high cost, time-consuming processes, and the inability to ensure complete biopsy sampling due to intratumoral heterogeneity. Indeed, biopsy samples are insufficient for molecular testing in approximately 4–10% of NSCLC patients [7,8,9]. Gene expression-based transcriptomic profiling, on the other hand, offers an approach that avoids additional invasive sampling that serves as an alternative or complementary method to tissue biopsy. Gene expression data can directly reveal the molecular identity of the tumor by reflecting changes in downstream signaling pathways caused by driver mutations [10]. With these characteristics, transcriptomic profiles constitute a valuable information source for determining mutation subtypes in samples with low tissue content, fine-needle aspiration biopsies, or serial clinical follow-ups.

The vast majority of current studies on machine learning-based NSCLC mutation classification are limited to a binary classification paradigm. This approach typically focuses on distinguishing the presence from the absence of a single driver mutation (particularly EGFR) and does not attempt to address three or more clinically meaningful classes simultaneously [10,11]. However, in a real clinical setting, a physician does not ask a single question: “Is this patient EGFR, KRAS, or TN?” This question must be answered simultaneously because each of these three classes corresponds to different treatment protocols, different prognoses, and different follow-up strategies. An EGFR-positive patient receives a TKI, a KRAS G12C-positive patient is a candidate for a KRAS inhibitor, and a TN patient requires evaluation of a combination of chemotherapy and immunotherapy.

A three-class driver mutation subtype classification—EGFR mutant, KRAS mutant, and TN—based on gene expression data is represented by an extremely rare study in the literature. Some of them [12] classified histological NSCLC subtypes using gene expression data; however, they did not address the EGFR/KRAS/TN driver mutation dimension. Another one [5] utilized information derived from protein–protein interaction networks in SVM-based cancer subtype classification; this approach requires mutation data and does not include batch effect correction at the transcriptomic level. There are also attempts at subclassification within KRAS-mutant LUAD in the literature; [13] used a consensus NMF clustering method on TCGA data to divide KRAS-mutant tumors into three biological subgroups (KL, KP, KC). However, this study is based on unsupervised statistical clustering and covers only KRAS-mutant tumors; it does not present a supervised multi-class machine learning approach that includes EGFR-mutant and TN subtypes. The authors of [12] also explicitly state that achieving true clinical benefit is difficult without overcoming the “current limitations in representing mutational heterogeneity in NSCLC subtype classification”. While another study [14] demonstrated the effectiveness of ensemble methods in general NSCLC classification, the authors’ study did not include stages such as multi-platform harmonization and independent external validation. These observations indicate that, to our knowledge, the three-class EGFR/KRAS/TN classification has been addressed using gene expression-based machine learning by very few studies; a closely related three-class formulation (EGFR-mutant, KRAS-mutant, and EGFR/KRAS-wild-type) was previously examined by Planck et al. [15] using differential-expression analysis and a centroid classifier (discussed in detail later in this section), but the present study differs in its modeling framework, cross-platform validation strategy, and interpretability methodology, highlighting a critical gap in the current literature.

A fundamental methodological challenge in multi-cohort gene expression studies is the batch effect that arises when integrating data from different platforms and laboratories. This technical variation masks the biological signal, severely limiting model generalizability [16]. Additionally, class imbalance—the disproportionate sample distribution among the EGFR, KRAS, and TN groups—leads standard classifiers to produce biased predictions for minority classes. The molecular and temporal heterogeneity of NSCLC significantly complicates the construction of stable prognostic models and the effective implementation of personalized therapy. Comprehensive studies such as TRACERx have demonstrated that driver mutations like EGFR and MET are typically clonal in origin—that is, widespread throughout the tumor—while late-emerging branch mutations drive therapeutic resistance and metastasis [17]. This finding highlights that NSCLC cases sharing the same diagnosis at the genetic level can exhibit distinct differences at the transcriptomic level, and that this variability directly influences treatment response. Machine learning-based heterogeneous ensemble frameworks offer a unique advantage in this context: complex, nonlinear mutational patterns that a single algorithm cannot capture can be consistently identified through the combination of multiple models derived from different learning paradigms [18,19]. In particular, a heterogeneous ensemble structure that combines gradient boosting (XGBoost, LightGBM, CatBoost), bagging (Random Forest), and kernel-based (SVM) algorithms possesses the capacity to model this mutational variation more comprehensively.

The interpretability of machine learning models is of critical importance for clinicians. For models to be used in clinical decision-support systems, they must not only provide high predictive accuracy but also clearly demonstrate which biological features they rely on to make decisions. This requirement is making the integration of explainable AI methods, such as SHapley Additive exPlanations (SHAP), into genomic classification studies increasingly important [11,20]. On the other hand, current NSCLC mutation prediction models are largely limited to single-center, single-platform studies with small sample sizes. It is well established that when data from different microarray or RNA-seq platforms are combined without correcting for cross-platform batch effects, technical variation can mask the biological signal. All these limitations strongly highlight the need for new approaches that address the following steps within an integrated pipeline: (1) multi-platform harmonization, (2) feature selection, (3) optimized heterogeneous ensemble learning, and (4) independent external validation.

The literature on machine learning applications for the classification of NSCLC subtypes has expanded significantly in recent years; however, existing studies differ from the approach taken in this study due to several fundamental limitations.

The largest body of literature on EGFR mutation prediction consists of radiomic and deep learning models based on CT imaging. A recent systematic review conducted by [10] (59 studies, 2018–2024 period) reveals that the median AUC value for radiomics–machine learning models was 0.78, while that for deep learning models was 0.84. However, only 29% of the studies included independent external validation; in external validation results, the average AUC drops significantly. This suggests that imaging-based models are over-optimized on single-center datasets. Furthermore, all of these approaches operate within a binary classification paradigm—EGFR mutant or wild-type—and do not incorporate a multi-class design encompassing KRAS and TN classes. The authors of [12] classified NSCLC histological subtypes using a neural network model trained on somatic mutation data and utilized SHAP for model decision analysis. However, this study focuses on distinguishing between adenocarcinoma and squamous-cell carcinoma; it does not address gene expression-based classification of EGFR/KRAS/TN driver mutations and does not include an independent external validation cohort. Another study [14], on the other hand, compared RF, SVM, XGBoost, and AdaBoost algorithms to demonstrate the superiority of ensemble methods in general NSCLC classification; however, the study does not include multi-platform harmonization or independent validation steps. One of the studies in the literature [21] analyzed multi-omic NSCLC data using consensus K-means clustering and highlighted tumor microenvironment features; however, this study adopts an exploratory clustering approach rather than supervised classification targeting driver mutations. DriverSub-SVM [5] performed patient-specific driver gene ranking by combining random walk and Bayesian Personalized Ranking methods on a protein–protein interaction network; it used the resulting scores as input for One-Against-One SVM in cancer subtype classification. This approach is limited in terms of clinical applicability due to its requirement for mutation data, lack of batch effect correction, and limited model interpretability. Ref. [11], on the other hand, examined XAI-based methods for feature selection across various cancer datasets and highlighted the importance of integrating interpretability tools into bioinformatics pipelines. In this study, the combined use of SHAP and Local Interpretable Model-agnostic Explanations (LIME) directly addresses this framework. Khadirnaikar et al. [21] applied consensus K-means clustering by compressing multi-omic NSCLC data into a low-dimensional space using machine learning and identified five molecular subgroups. While this study demonstrates the strengths of multi-omic integration, it has a limited design as it uses only RNA-seq data and lacks independent external validation. Almufareh et al. [6] developed a deep neural network using DNA methylation profiles for the histological classification of LUAD and LUSC and reported an accuracy exceeding 99%; however, the study focuses on histological classification rather than mutation subtype differentiation. Li et al. [14] compared RF, SVM, XGBoost, and AdaBoost algorithms to molecularly cluster stage 3–4 NSCLC patients; they reported that immune-associated signatures overlapped with the KRAS mutant group. However, the study was single-platform and batch effects were not addressed. Compared to the existing studies summarized in Table 1, this study represents one of the few that simultaneously incorporates the following features within a single pipeline: (a) gene expression-based three-class driver mutation differentiation, (b) multi-platform batch effect correction, (c) SHAP-based feature selection and interpretability, (d) Bayesian-optimized heterogeneous ensemble learning, and (e) cross-platform external validation on an independent RNA-seq cohort. It should be noted that each of these components (ComBat harmonization, SHAP-based feature selection, Bayesian hyperparameter optimization, and heterogeneous ensembling) is an established method in its own right; the contribution of the present study lies not in proposing a new algorithm, but in their systematic integration, a leak-aware cross-validation design in which a specific, acknowledged source of residual information leakage (SHAP-based feature selection fixed across outer folds, Section 2.5) was identified and its impact quantitatively bounded via ablation, and empirical benchmarking for the specific, underexplored three-class EGFR/KRAS/TN classification setting. It should be explicitly noted that the three-class EGFR/KRAS/wild-type problem addressed here is conceptually related to the classification scheme introduced by Planck et al. [15], who developed a 96-gene expression signature distinguishing EGFR-mutant, KRAS-mutant, and EGFR/KRAS double-wild-type lung adenocarcinoma using gene expression profiles from 914 lung adenocarcinomas assembled from multiple independent microarray cohorts, including GSE31210 and GSE13213, which are also used as internal-cohort components in the present study. The TN class defined here, based on EGFR/KRAS mutual exclusivity, is therefore conceptually similar to Planck et al.’s EGFR/KRAS double-wild-type group, and this overlap in both the classification target and a subset of the underlying data is acknowledged rather than obscured. However, three concrete distinctions separate the present work from Planck et al.’s study. First, and most importantly, all cohorts used by Planck et al. for discovery and validation were microarray-based (Affymetrix, Agilent, Illumina, or custom cDNA platforms); no RNA-seq validation was performed, since RNA-seq was not yet a standard clinical transcriptomic platform in 2013. The present study instead performs external validation across a genuinely different sequencing technology (microarray-trained model evaluated on RNA-seq-derived TCGA-LUAD data), a cross-platform generalization problem that did not exist in the same form at the time of Planck et al.’s publication and that requires dedicated harmonization methodology (Section 2.4). Second, Planck et al.’s classifier is built on univariate differential expression analysis (ANOVA) followed by unsupervised hierarchical clustering to derive a fixed gene signature, whereas the present pipeline employs a Bayesian-optimized heterogeneous ensemble of five machine learning classifiers with SHAP-driven, model-based feature selection and NSGA-II multi-objective ensemble weighting. Third, this study provides model-level explainability (SHAP beeswarm analysis, Section 3.6) and pathway-level biological validation (Reactome and Gene Ontology enrichment) of the selected features, which were not part of the original Planck et al. methodology. The partial data overlap (GSE31210, GSE13213) is limited to two of the three internal-cohort GEO datasets and does not extend to the TCGA-LUAD external validation cohort, which was not part of Planck et al.’s study.

Beyond the use of different computational tools, this study contributes four concrete insights not established by Planck et al. or other prior work summarized in Table 1. First, a systematic six-configuration ablation analysis (Section 3.5) quantifies the individual contribution of each pipeline component (batch harmonization, class balancing, feature selection, hyperparameter optimization, and ensembling) to overall classification performance in this three-class setting, providing a reusable methodological benchmark for future studies. Second, the comparative evaluation of ParetoVoting against fixed-weight SoftVoting yields an honest negative methodological finding: multi-objective NSGA-II ensemble weighting provides no measurable benefit over simple equal-weight averaging in this setting (Cliff’s δ=−0.12), a result that tempers the common assumption that more sophisticated ensemble weighting necessarily improves performance. Third, this study provides a quantitative, cross-platform characterization of the transcriptomic overlap between KRAS-mutated and EGFR/KRAS-wild-type (TN) tumors, extending Planck et al.’s qualitative observation of this same difficulty within single-platform microarray data into an explicit, RNA-seq-validated confusion-matrix characterization (Section 2.6.6) that links classification errors to a specific, permissive decision threshold. Fourth, the independent recovery of DUSP4 as a top discriminative feature by SHAP-based analysis, despite using an entirely different feature-selection paradigm and a partially non-overlapping cohort set relative to Planck et al.’s ANOVA-based signature, provides convergent, methodology-independent evidence for the biological relevance of this gene in EGFR/KRAS/TN differentiation.

## 2. Materials and Methods

### 2.1. Datasets

Three independent LUAD microarray datasets were obtained from the GEO (Gene Expression Omnibus) database: GSE31210 (Affymetrix GPL570, n=226; [22]), GSE13213 (Agilent GPL6480, n=117; [23]), and GSE72094 (Affymetrix GPL15048, n=442; NCBI GEO, 2014) [24]. ALK fusion-positive samples within GSE31210 (n=11) were excluded due to insufficient cohort size for reliable model training. After exclusion, the combined internal cohort comprises 774 samples across three driver mutation subtypes: EGFR mutant (n=219), KRAS mutant (n=189), and triple-negative (TN, n=366). TCGA-LUAD was selected as the external cohort because it is among the largest publicly available lung adenocarcinoma RNA-seq cohorts with matched somatic mutation calls (MC3), it represents a distinct sequencing technology from the microarray-based internal cohorts—directly testing cross-platform generalization—and it is histologically matched to LUAD, avoiding confounding by histological subtype. Accordingly, all conclusions in this study are scoped to lung adenocarcinoma and should not be generalized to other NSCLC histological subtypes without independent validation. RNA-seq gene expression profiles were obtained from the UCSC Xena platform (HiSeqV2, log2(RPKM+1)); somatic mutation labels were derived from the MC3 mutation file by applying EGFR/KRAS mutual exclusivity.

TN was defined via EGFR/KRAS mutual exclusivity in all cohorts; ALK-negativity is additionally confirmed only for GSE31210 (n=11 excluded), not for GSE13213, GSE72094, or TCGA-LUAD. We retain the term “TN” (triple-negative) for consistency with related driver-mutation classification literature, though the classification is technically based on EGFR/KRAS dual-negativity rather than three independently confirmed negative markers; this should be interpreted accordingly throughout this manuscript.

**Figure 1:**
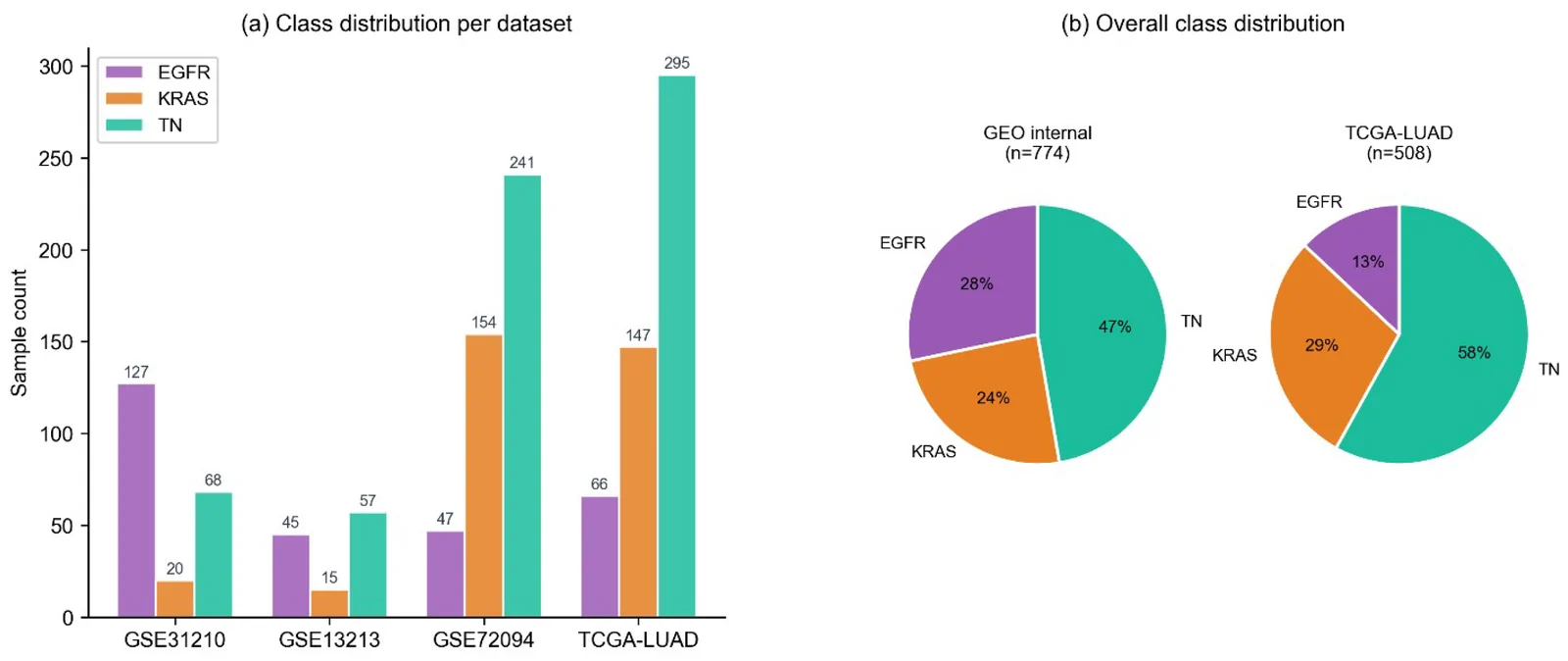
Distribution of datasets and class imbalance across cohorts. (**a**) Sample distribution per cohort stratified by driver mutation subtype. GSE72094 shows enrichment of KRAS-mutant cases compared to other GEO datasets. (**b**) Observed (pre-SMOTE) class distribution in the internal GEO cohort (n=774) and TCGA-LUAD external validation cohort (n=508). This class imbalance was subsequently addressed via SMOTE oversampling applied only to the training partition within each cross-validation fold (Section 2.6.2); the distribution shown here reflects the raw, unmodified cohort composition.

The dataset characteristics are summarized in Table 2, and the class distribution across cohorts is illustrated in Figure 1.

### 2.2. Preprocessing and Feature Filtering

Probe-to-gene mapping was performed using platform-specific annotation files (GPL570, GPL6480, GPL15048). Expression values were averaged for probes representing the same gene. The intersection of the three GEO cohorts resulted in 16,156 common genes. A total of 132,303 missing values resulting from probe-gene mapping inconsistencies were imputed using median imputation on a gene-by-gene basis. Subsequently, 2000 genes with the highest inter-sample variance were selected for dimension reduction and to preserve the most informative features. Since the high dimensionality of microarray data remains a fundamental challenge threatening classification validity, the systematic narrowing down of genes contributing to classification is of great importance [25]. Median imputation and variance-based gene pre-filtering (top 2000 genes) were performed once on the pooled cohort prior to the outer cross-validation split, rather than per-fold. This is an additional, unquantified source of the same optimistic-bias risk already acknowledged and bounded for SHAP feature selection (Section 2.5, Table 3); we expect its magnitude to be smaller, since it precedes any model-based decision, but we did not separately measure it in the present study.

### 2.3. Basic Classifiers

Five basic classifiers were used in the heterogeneous ensemble structure. XGBoost, based on gradient boosting, was included in the pipeline due to its superior performance in high-dimensional gene expression data and resistance to over-learning [26]. LightGBM provides speed and memory efficiency with its gradient-based one-sided sampling strategy [27]. CatBoost offers unbiased gradient estimation with sequential boosting [28]. Random Forest combines decision tree ensembles created by bootstrap sampling and random feature selection through voting [29]. SVM is a kernel-based classifier that maximizes the separation between classes with the maximum margin hyperplane principle [30].

### 2.4. Batch Effect Correction (ComBat)

To address technical variations arising from different microarray platforms, empirical Bayesian harmonization based on the empirical Bayesian framework ComBat has been implemented in Python via the neuroCombat library [16,31]. Empirical Bayesian correction provides an effective solution to this problem in biomarker studies containing a reference batch [32]. Each GEO dataset was defined as a separate batch. The observed gene expression value for the jth sample in the ith batch for gene g is modeled as follows:

(1) $$Y_{i,j,g}=\alpha_{g}+X\beta_{g}+\gamma_{i,g}+\delta_{i,g}\;\epsilon_{i,j,g}$$

**Note:** $Y_{i,j,g}$: expression value; $\alpha_{g}$: gene-specific general mean; $X\beta_{g}$: biological covariate effects; $\gamma_{i,g}$: additive batch effect; $\delta_{i,g}$: multiplicative batch effect; $\epsilon_{i,j,g}∼N\left(0,\sigma_{g}^{2}\right)$.

Batch-corrected values are obtained using empirical Bayesian minimization estimates:

(2) $$Y_{i,j,g}^{*}=\frac{Y_{i,j,g}-\alpha_{g}-X\beta_{g}-\gamma_{i,g}^{*}}{\delta_{i,g}^{*}}+\alpha_{g}+X\beta_{g}$$

**Note:** Asterisks denote empirical Bayes shrinkage estimates. For external validation, cross-platform harmonization was performed using a reference-batch ComBat approach (ref_batch = 0), in which batch correction parameters were estimated exclusively from the GEO training cohort and subsequently applied to the TCGA-LUAD cohort. For the final external-validation model, the gene universe was restricted to genes shared between the internal cohort and TCGA-LUAD prior to ComBat harmonization and gene selection so that the same harmonization parameters could be applied consistently to both cohorts. This restriction is based solely on gene identity (which genes are measured on each platform) and does not draw on any TCGA sample values or labels. This procedure ensures that the external validation set remains entirely independent of the training preprocessing pipeline, in accordance with the principles of unbiased external validation.

The effectiveness of ComBat harmonization was visually validated using PCA and t-SNE (Section 3.1). Prior to correction, PC1 (11.5% variance) reflected platform-specific variation; GSE72094 was distinctly separated from the other two cohorts. After applying ComBat, platform-induced clustering disappeared, and samples were reorganized according to biological mutation classes (EGFR, KRAS, TN). ComBat batch-correction parameters (Section 2.4) were estimated once on the full internal cohort prior to the 5-fold split, allowing held-out test samples to contribute to the estimated parameters within each fold. To quantify the resulting bias, ComBat, SHAP feature selection, and hyperparameter tuning were all re-derived using only one held-out fold’s (fold 2/5) training partition, via the same reference-batch technique used for external validation. This yielded F1-macro = 0.707 (vs. 0.726 for the fixed pipeline on this fold; ΔF1 = −0.019), indicating a real but moderate bias; a full five-fold reconstruction was not performed due to its computational cost. This bias does not affect the external validation below (Section 3.3), where ComBat parameters were estimated exclusively from the internal training cohort and applied to TCGA-LUAD without any contribution from external samples (Table 3).

### 2.5. SHAP-Based Feature Selection

Feature selection was performed using SHAP [33] values obtained from a Random Forest classifier (n_estimators=200, max_depth=10, class_weight=balanced) trained on the training set of the first outer (internal) cross-validation fold. The SHAP value for the *i*-th characteristic of the *j*-th sample is calculated as follows:

(3) $$\phi_{i}\left(f,x_{j}\right)=\sum_{S\subseteq F\setminus \{i\}}\frac{|S|!\;(|F|-|S|-1)!}{|F|!}\left[f(S\cup \{i\})-f(S)\right]$$

**Note:** *F* denotes the full feature set; *S* is a subset of features excluding feature *i*; f(S) represents the model prediction using subset *S*. This formulation distributes the marginal contribution of each feature across all possible feature coalitions via a weighted average.

Global feature importance is calculated as the average absolute SHAP value across all examples and classes:

(4) $$I_{i}=\frac{1}{nK}\sum_{k=1}^{K}\sum_{j=1}^{n}\left|\phi_{i}^{\left(k\right)}\left(f,x_{j}\right)\right|$$

**Note:** *n* denotes the number of samples and *K* = 3 corresponds to the number of classes (EGFR, KRAS, TN). $\phi_{i}^{\left(k\right)}$ represents the SHAP value of feature *i* for class *k*. The top 500 genes ranked by $I_{i}$ were selected for model training. To prevent data leakage, SHAP-based feature selection was performed on the training set of the first outer (internal) cross-validation fold, and the same gene set was consistently applied across all subsequent folds and the TCGA-LUAD external validation cohort. Although feature selection was performed on the training partition of the first outer fold and applied consistently across all subsequent folds, this represents a deliberate methodological choice to ensure reproducibility and reduce computational overhead, consistent with established practice in high-dimensional transcriptomic classification studies [25,34]. This design should be explicitly acknowledged as carrying a mild data leakage risk: because the same 500-gene set (selected using outer fold 1’s training partition) is subsequently used to featurize the test partitions of outer folds 2–5, those four folds are not fully independent of the fold-1 training data at the feature-selection stage, even though model fitting and evaluation within each fold remain leak-free. To assess potential optimistic bias introduced by this design, we conducted an ablation experiment in which all 2000 variance-selected genes were used without SHAP filtering. The resulting decline in F1-macro (ΔF1 = −0.018; see the ablation analysis in Section 3.5) confirms that SHAP-based feature selection contributes genuine predictive value rather than artificial inflation of performance estimates, and the magnitude of this decline is small relative to the overall performance range across ablation configurations, suggesting that any residual leakage effect is limited. Furthermore, the consistency of SHAP-selected gene sets across folds—with core discriminative genes such as DUSP4, CYP24A1, and KRAS appearing prominently across all cross-validation iterations—provides additional empirical support for the stability of the selected feature space.

### 2.6. Model Development Framework

**Figure 2:**
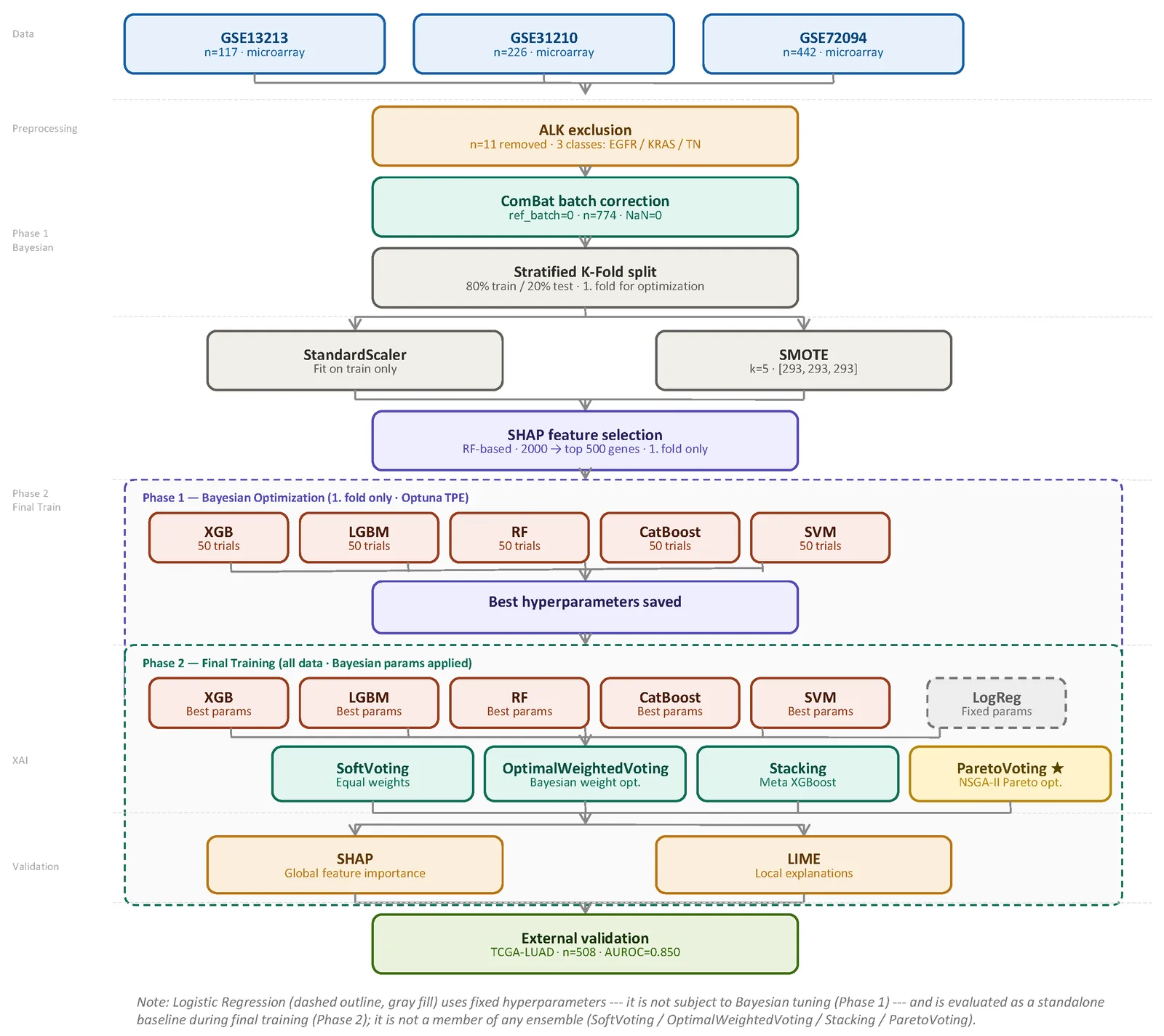
Overall analysis pipeline from multi-cohort data integration to external validation. Hyperparameter optimization, SHAP-based feature selection, and ensemble-weight selection (Section 2.6.1) used only the training partition of the first outer fold; the test partition of that fold was never accessed for these steps.

The proposed methodology integrates multi-cohort data integration, ComBat harmonization, SHAP-based feature selection, Bayesian-optimized heterogeneous ensemble learning, and independent external validation steps into a single integrated pipeline. The overall workflow encompassing all these steps is presented in Figure 2.

#### 2.6.1. Cross-Validation Design and Prevention of Data Leakage

To reduce the risk of data leakage—which is particularly critical in multi-step pipelines—the following principles were adopted: (1) Feature scaling (StandardScaler) was applied only to the training set of each fold; the test set was not included in this step. (2) SMOTE sampling was applied only to the training set of each fold; the test set was never oversampled, and evaluation was performed on the original class distribution. (3) Bayesian hyperparameter optimization was performed only on the training set of the first outer fold; the optimal parameters were fixed and applied uniformly to all five folds. (4) Ensemble weight optimization was performed using an inner cross-validation split of the first fold’s training partition; the resulting weights were applied as fixed coefficients to all subsequent folds. Two exceptions to this per-fold design should be noted explicitly: SHAP-based feature selection, and median imputation and variance-based gene pre-filtering were each performed once, on the pooled cohort or on the first fold’s training partition, and then reused across all folds. These represent an acknowledged, partially unquantified source of optimistic bias, bounded where feasible by the leak-free sensitivity analysis.

#### 2.6.2. SMOTE Balanced Sampling

To address class imbalance, SMOTE (Synthetic Minority Over-sampling Technique; [35]) was applied to the training set of each fold (EGFR: 28%, KRAS: 24%, TN: 47%). SMOTE generates synthetic samples via linear interpolation between a minority-class sample and one of its k nearest neighbors:

(5) $$x_{\mathrm{new}}=x_{i}+\lambda \left(x_{i(\mathrm{NN})}-x_{i}\right),\;\lambda ∼U\left(0,1\right)$$

**Note:** $x_{i}$ denotes a selected minority class sample; $x_{i(\mathrm{NN})}$ represents one of its *k* nearest neighbors (k=5); λ is a random scaling factor in the interval [0,1]. SMOTE was applied after standardization using StandardScaler to prevent scale-dependent artifacts.

#### 2.6.3. Bayesian Hyperparameter Optimization

A two-stage optimization strategy was applied. In the first stage, a separate Bayesian optimization script was run using the Optuna framework with 50 trials per model; global optimal hyperparameters were determined for XGBoost, LightGBM, Random Forest, and CatBoost. The obtained parameters were integrated into the final pipeline as fixed values to ensure consistency and reproducibility during the 5-fold CV and external validation stages. For SVM, hyperparameter optimization was performed within the final pipeline via a separate Bayesian run on the first outer (internal) cross-validation fold. The TPE classifier models the gain function as follows:

(6) $$\mathrm{EI}\left(x\right)\propto \frac{\gamma \;l(x)}{\left(1-\gamma )\;g(x\right)}$$

**Note:** l(x) denotes the density of configurations yielding high objective values; g(x) represents the density of poorly performing configurations; γ corresponds to the top 25% quantile. The optimization objective is the macro-average F1 score evaluated using 3-fold cross-validation on the training set of the first outer (internal) cross-validation fold.

(7) $$\theta^{*}=\arg\max_{\theta }\frac{1}{K_{\mathrm{ic}}}\sum_{k=1}^{K_{\mathrm{ic}}}F1_{\mathrm{macro}}\;\left(y_{\mathrm{validation}}^{\left(k\right)},y_{\mathrm{prediction}}^{\left(k\right)}\left(\theta \right)\right)$$

**Note:** $K_{\mathrm{ic}}=3$ denotes the number of cross-validation folds and θ represents the hyperparameter vector. The optimized hyperparameters for all classifiers are summarized in Table 4.

The tuning protocol differs by design: XGBoost, LightGBM, Random Forest, and CatBoost were tuned via a separate 50-trial Optuna run (Table 4), with parameters fixed across folds; SVM was tuned within the pipeline using an equivalent 50-trial budget on the first fold. Logistic Regression instead uses fixed, minimally-tuned hyperparameters, reflecting its role as a standard interpretable baseline rather than a competitively-optimized model; its reported F1-macro = 0.642 (Table 5) should therefore be read as a baseline floor. The Elastic-Net, Nearest-Centroid, and Linear-SVM baselines added in Table 5 were tuned under a consistent, lightweight protocol and offer a fairer basis for judging whether the ensemble’s added complexity is justified.

#### 2.6.4. Heterogeneous Ensemble Structure and Soft Voting

A heterogeneous ensemble consisting of five base classifiers from different learning paradigms was constructed: XGBoost, LightGBM, and CatBoost (gradient boosting), Random Forest (bagging), and SVM (kernel-based, maximum margin classifier). Success in ensemble learning depends not only on the individual accuracy of the base models but also on the diversity among them [18,19]. In the context of LUAD mutational heterogeneity, complex nonlinear patterns that a single learning paradigm cannot capture are modeled more comprehensively with this heterogeneous structure. The primary ensemble strategy is Soft Voting, which combines class probability outputs using an equally weighted average:

(8) $$P_{\mathrm{SV}}\left(y=c|x\right)=\frac{1}{M}\sum_{m=1}^{M}P_{m}\left(y=c|x\right)$$

**Note:** M=5 denotes the number of base classifiers (XGBoost, LightGBM, Random Forest, CatBoost, and SVM). $P_{m}\left(y=c|x\right)$ represents the predicted probability for class *c* from the *m*-th model, where c∈{EGFR,KRAS,TN}. The final prediction is obtained as follows:

$$\hat{y}=\arg\max_{c}P_{\mathrm{SV}}\left(y=c|x\right).$$

An ablation study confirmed SVM’s contribution to ensemble diversity: removing SVM reduced the F1-macro score from 0.735 to 0.722; performance converged to the level of a single XGBoost model (F1 = 0.723). This finding demonstrates that SVMs generate complementary decision boundaries with gradient boosting models. For comparison, the Bayesian-optimized weighted Optimal Voting (OptimalVoting) and the meta-learning-based Stacking classifier were also evaluated:

(9) $$P_{\mathrm{OV}}\left(y=c|x\right)=\frac{\sum_{m=1}^{M}w_{m}\;P_{m}\left(y=c|x\right)}{\sum_{m=1}^{M}w_{m}}$$

(10) $$w_{m}^{*}=\arg\max_{w_{m}}\;F1_{\mathrm{macro}}\left(y_{\mathrm{validation}},y_{\mathrm{prediction}}\left(w_{m}\right)\right),\;w_{m}\in \left[0.1,5.0\right]$$

**Note:** $P_{\mathrm{OV}}\left(y=c|x\right)$ denotes the weighted ensemble probability for class *c*. $w_{m}$ represents the optimized weight assigned to the *m*-th base classifier. The weights were tuned by maximizing the mean macro-average F1 score across an inner 3-fold cross-validation performed strictly within the training partition of the first outer fold, under the constraint $w_{m}\in \left[0.1,5.0\right]$. To avoid information leakage, the outer test set was never used for weight selection, and the resulting fixed weights were applied consistently across all cross-validation folds.

In the stacking classifier, an XGBoost meta-learner ($n_{\mathrm{estimators}}=200$, $max_{\mathrm{depth}}=3$, lr=0.05, passthrough=True) was used; the meta-feature matrix combines the base model probability outputs with the original 500 genes:

(11) $$Z=\left[P_{1}\left(x\right),P_{2}\left(x\right),\ldots ,P_{M}\left(x\right),x\right]\in R^{n\times (MK+d)}$$

**Note:** *Z* denotes the meta-feature matrix constructed for the stacking classifier. $P_{m}\left(x\right)$ represents the class probability vector predicted by the *m*-th base model, each of dimension *K*. d=500 corresponds to the number of original gene expression features, K=3 is the number of classes, and M=5 denotes the number of base models.

#### 2.6.5. ParetoVoting: NSGA-II Multi-Objective Ensemble Weight Optimization

Unlike Optimal Voting, in which a single global weight vector was derived once on the first outer fold and subsequently held fixed, the ParetoVoting ensemble re-optimized its weight vector independently within each of the five outer cross-validation folds, using a nested (inner) 3-fold cross-validation scheme applied strictly to that fold’s training partition. This design avoids any leakage from the outer test set into weight selection while allowing the ensemble to adapt to fold-specific class balance and model behavior.

Within each outer fold, base classifiers (XGBoost, LightGBM, Random Forest, CatBoost, SVM) were retrained on each inner-fold training split, and their held-out inner-fold probabilities were combined using the weighted-voting rule of Equation (9). Two objectives were optimized simultaneously:

(12) $$\max_{w}\;\left({\bar{F1}}_{\mathrm{macro}}\left(w\right),\;\bar{\mathrm{MCC}}\left(w\right)\right)$$

**Note:** ${\bar{F1}}_{\mathrm{macro}}\left(w\right)$ and $\bar{\mathrm{MCC}}\left(w\right)$ denote the macro-F1 and Matthews Correlation Coefficient, respectively, averaged over the $K_{\mathrm{inner}}=3$ inner cross-validation folds, for a given weight vector $w=(w_{\mathrm{xgb}},w_{\mathrm{lgbm}},w_{\mathrm{rf}},w_{\mathrm{cat}},w_{\mathrm{svm}})$, subject to $w_{\mathrm{xgb}},w_{\mathrm{lgbm}},w_{\mathrm{cat}},w_{\mathrm{svm}}\in \left[0.1,\;5.0\right]$ and $w_{\mathrm{rf}}\in \left[0.1,\;3.0\right]$.

The multi-objective search was performed using the NSGA-II algorithm (Optuna NSGAIISampler), evaluating 200 trials per outer fold with the fold-specific random seed 42+f (where *f* denotes the outer fold index, f=0,…,4). This yielded, for each outer fold, a Pareto-optimal front of non-dominated weight vectors representing trade-offs between macro-F1 and MCC. The final weight vector for that fold was selected as the point on the Pareto front maximizing the unweighted sum of both objectives:

(13) $$w_{f}^{*}=\arg\max_{w\;\in \;{\mathrm{ParetoFront}}_{f}}\left[{\bar{F1}}_{\mathrm{macro}}\left(w\right)+\bar{\mathrm{MCC}}\left(w\right)\right]$$

**Note:** ${\mathrm{ParetoFront}}_{f}$ denotes the set of non-dominated weight vectors obtained for outer fold *f*. The resulting fold-specific weight vector $w_{f}^{*}$ was then applied to the corresponding outer-fold test-set probabilities to generate the ParetoVoting prediction for that fold, following Equation (9).

Because ParetoVoting weights were re-optimized independently within each outer fold rather than fixed globally, no single ParetoVoting weight vector exists that could be transferred directly to the independent TCGA-LUAD external validation cohort; doing so would require either an arbitrary choice among the five fold-specific weight vectors or a separate NSGA-II optimization on the full internal cohort, introducing an additional, unvalidated design decision. Given that ParetoVoting’s internal cross-validation performance was statistically indistinguishable from that of the fixed-weight SoftVoting ensemble (F1-macro =0.7364 vs. 0.7350; Cliff’s δ=−0.12, negligible; Table 6), the fixed-weight SoftVoting ensemble was selected for external validation, as described in Section 3.3.

#### 2.6.6. Class-Based Probability Threshold Optimization

A systematic shift in probability distribution is observed between the microarray-trained model and the RNA-seq TCGA cohort; this is a result of cross-platform effect alignment. To prevent information leakage, class-based decision thresholds were optimized on an internal validation subset derived from the training cohort, ensuring that the external TCGA-LUAD cohort remained entirely unseen during threshold calibration. Specifically, this subset was obtained by applying a single stratified 5-fold split (independent of, and not to be confused with, the five outer cross-validation folds used for model evaluation in Section 2.6.1) to the full internal cohort (n=774, post-ALK-exclusion, ComBat-harmonized, SHAP-selected feature space); the first fold’s validation partition (approximately 20% of the internal cohort, n≈155) served as the internal validation subset, while base classifiers used for threshold calibration were trained on the corresponding 80% training partition of this same split. The optimal thresholds obtained were: EGFR = 0.30, KRAS = 0.15, TN = 0.34.

(14) $$t_{c}^{*}=\arg\max_{t\in [0.1,\;0.9]}\;F1\left(y_{c},\;I\;\left[P(y=c|x)\geq t\right]\right)$$

**Note:** *t* denotes the decision threshold scanned in increments of 0.01; I[·] is the indicator function; F1 represents the class-specific binary F1 score.

(15) $$\hat{y}=\arg\max_{c\in \{\mathrm{EGFR},\;\mathrm{KRAS},\;\mathrm{TN}\}}\frac{P(y=c|x)}{t_{c}^{*}}$$

**Note:** This normalization corrects for platform-induced systematic bias. Threshold optimization improved the macro-average F1 score from 0.673 to 0.700. Gene symbols were matched directly between TCGA and GEO (no additional probe mapping needed for RNA-seq). Of the 500 SHAP-selected genes, 496 were present in the TCGA dataset after harmonization; the remaining four genes (0.8%—PRSS2, MUC5AC, TUBB4B, and SPTSSA) were imputed with the training-cohort mean expression value for that gene, rather than zero, to avoid introducing an artificial, class-biased signal. These four genes ranked 82/500, 150/500, 322/500, and 393/500, respectively, by SHAP importance; PRSS2 and MUC5AC fall within the upper half of the selected feature set; so, training-mean imputation is an important safeguard for these genes specifically, rather than a negligible detail.

#### 2.6.7. Evaluation Metrics

Model performance was evaluated using accuracy, macro-average F1, Matthews Correlation Coefficient (MCC), and macro-average Area Under the Receiver Operating Characteristic curve (AUROC) (one-versus-all strategy). MCC is known as a more informative performance metric compared to accuracy and F1 metrics in unbalanced class distributions [36]; it was adopted as the primary metric in this study due to its robustness in multi-class unbalanced conditions:

(16) $$\mathrm{MCC}=\frac{\sum_{k}\sum_{l}\sum_{m}\left(C_{kk}C_{lm}-C_{kl}C_{mk}\right)}{\sqrt{\left(\sum_{k}T_{k}\right)\left(\sum_{k}P_{k}\right)}}$$

**Note:** *C* denotes the K×K confusion matrix, where $C_{kl}$ represents the number of samples belonging to class *k* predicted as class *l*. Indices *k*, *l*, and *m* denote class labels. MCC ranges from −1 (complete disagreement) to +1 (perfect prediction), with 0 indicating random performance.

(17) $$F1_{\mathrm{macro}}=\frac{1}{K}\sum_{c=1}^{K}\frac{2\cdot {\mathrm{Precision}}_{c}\cdot {\mathrm{Sensitivity}}_{c}}{{\mathrm{Precision}}_{c}+{\mathrm{Sensitivity}}_{c}}$$

**Note:** K=3 denotes the number of classes. ${\mathrm{Precision}}_{c}=\frac{TP_{c}}{TP_{c}+FP_{c}}$ and ${\mathrm{Sensitivity}}_{c}=\frac{TP_{c}}{TP_{c}+FN_{c}}$ represent the precision and recall for class *c*, respectively.

The overall differences in model performance were assessed using the Friedman test. For pairwise comparisons, effect sizes were reported using Cliff’s Delta (δ), a non-parametric measure independent of sample size. Effect size interpretation followed standard thresholds: δ<0.147 (negligible), δ<0.330 (small), δ<0.474 (moderate), and δ≥0.474 (large).

#### 2.6.8. Explainability with SHAP and LIME

Global explainability was assessed using SHAP beeswarm plots computed on the XGBoost component of the SoftVoting ensemble, applied to the held-out test set of the final cross-validation fold [20]. Local explanations were generated using LIME [37] with num_features=15 for representative samples from each class.

#### 2.6.9. Application Details

All analyses were performed using Python 3.10. The following libraries were used: scikit-learn 1.3, XGBoost 2.0, LightGBM 4.0, CatBoost 1.2, Optuna 3.3, imbalanced-learn 0.11 (SMOTE), neuroCombat 0.0.7, SHAP 0.44, pandas 2.0, and numpy 1.24. GPU-accelerated training was enabled where supported (XGBoost: tree_method = hist, device = cuda; LightGBM: device = gpu; CatBoost: task_type = GPU) and performed on an NVIDIA GeForce RTX 4080 GPU.

To ensure reproducibility, the random seed was fixed at 42 across all preprocessing, training, and evaluation stages.

### 2.7. Use of AI Tools

Large language model assistance (Claude, Anthropic) was used during manuscript preparation for language editing, text structuring, and code development support. All scientific content, methodology, results, and conclusions were independently developed and validated by the authors.

## 3. Results

### 3.1. ComBat Harmonization—Visual Validation

**Figure 3:**
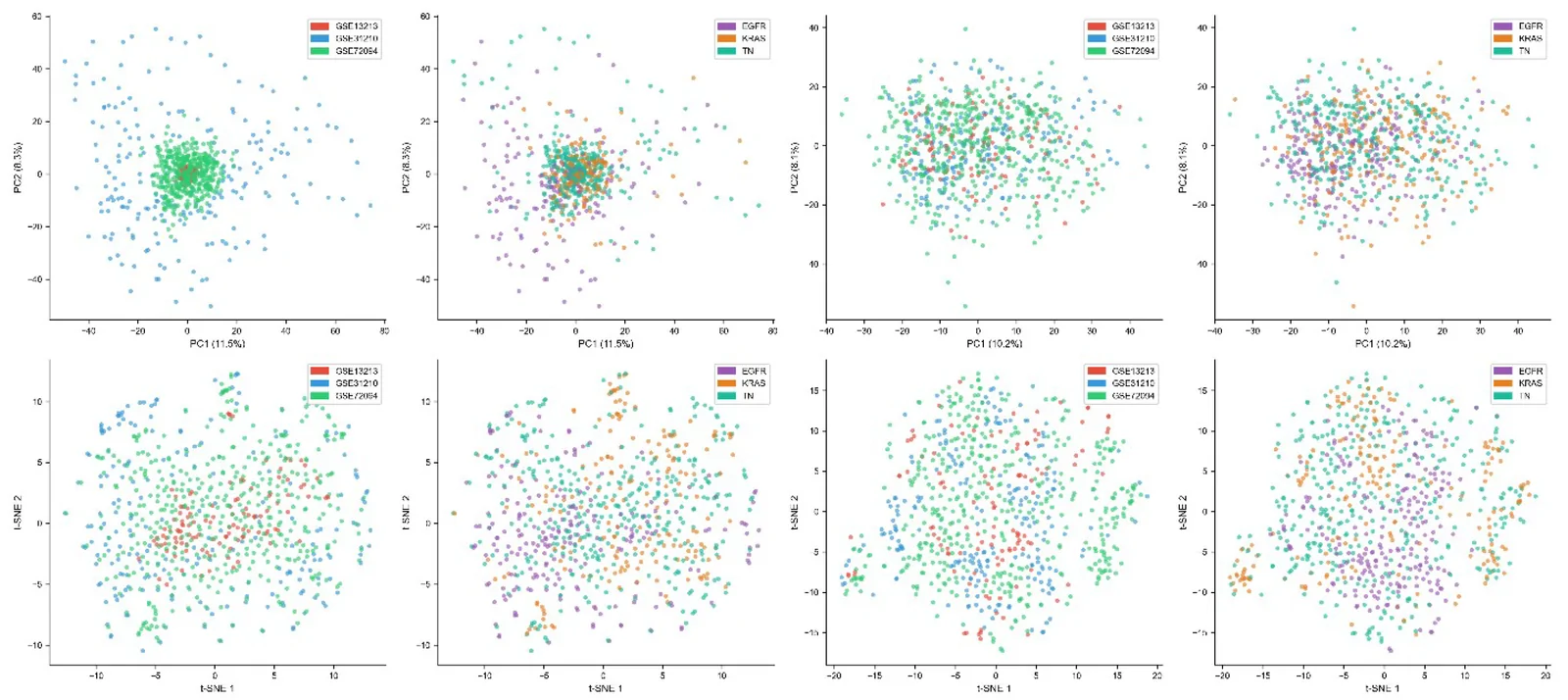
PCA (**top**) and t-SNE (**bottom**) analysis before and after ComBat harmonization (n=774). The left panels illustrate cohort origin, whereas the right panels represent mutation subtype distribution. EGFR samples are shown in purple, KRAS in orange, and TN in green.

The effectiveness of ComBat harmonization was evaluated using PCA and t-SNE dimension reduction methods (Figure 3). Before correction, PC1, which explained 11.5% of the total variance, distinguished samples based on platform origin; the GSE72094 cohort was distinctly separated from the other two cohorts. Similarly, in the t-SNE analysis, samples clustered according to platform identity. After applying ComBat, platform-induced clustering disappeared, and samples were redistributed according to biological mutation classes (EGFR, KRAS, TN). The decrease in PC1 variance from 11.5% to 10.2% numerically confirms that the dominant batch component was successfully removed. These findings demonstrate that ComBat effectively eliminates cross-platform technical variation while preserving the biologically meaningful gene expression pattern.

### 3.2. Results of 5-Fold Cross-Validation

The results of the five-fold stratified cross-validation are summarized in Table 5 (see also Figure S2 for a visual comparison). The ParetoVoting ensemble demonstrated the highest performance across all metrics (F1-macro = 0.7364 ± 0.023, Accuracy = 0.7365, MCC = 0.5826, AUROC = 0.8772), marginally ahead of the fixed-weight SoftVoting ensemble (F1-macro = 0.7350 ± 0.036, Accuracy = 0.7352, MCC = 0.5805, AUROC = 0.8773); as detailed in Table 6, this difference is not statistically distinguishable (Wilcoxon p=1.000, paired 95% CI =[−0.021,0.018], Cliff’s δ=−0.12, negligible). Given this statistical equivalence and its simpler, fixed-weight design, SoftVoting was carried forward for independent external validation (Section 3.3). Ensemble methods consistently outperformed single classifiers. Among the individual models, XGBoost demonstrated the highest performance (F1-macro = 0.7226 ± 0.038); SoftVoting outperformed it by 1.7%. Logistic Regression exhibited the lowest performance (F1-macro = 0.6417 ± 0.038). To address whether the proposed ensemble’s added complexity is justified, three additional simple, interpretable baselines were evaluated on the identical 5-fold splits and feature space: elastic-net logistic regression (F1-macro = 0.630), nearest-centroid classification (F1-macro = 0.574), and a linear-kernel SVM (F1-macro = 0.583). All three baselines, along with multinomial logistic regression already reported above (F1-macro = 0.642), perform substantially below the primary ensemble (ParetoVoting, F1-macro = 0.736; SoftVoting, F1-macro = 0.735), by a margin of approximately 10 percentage points in macro-F1. This gap is not “slight”; it indicates that the heterogeneous ensemble architecture, combined with SHAP-based feature selection and Bayesian hyperparameter optimization, captures discriminative structure that simple linear or centroid-based models on the same feature space cannot, supporting the additional modeling complexity adopted in this study. Class-based F1 scores for both the primary ParetoVoting ensemble and the externally-validated SoftVoting ensemble are presented in Table 7. For ParetoVoting, the EGFR subtype achieved the highest class-based F1 score (0.756 ± 0.038), followed by TN (0.733 ± 0.025) and KRAS (0.720 ± 0.051); SoftVoting showed a closely comparable pattern (EGFR 0.756 ± 0.048, TN 0.732 ± 0.033, KRAS 0.717 ± 0.061), with negligible differences between the two ensembles across all three classes. The relatively lower KRAS performance in both ensembles aligns with KRAS being represented by a smaller number of samples in the internal cohort (n=189).

The Friedman test confirmed that there is a statistically significant overall difference between model performances ($\chi^{2}=23.79$, p=0.0046). The paired Wilcoxon signed-rank test and 95% confidence intervals (Table 6) show that the SoftVoting-vs-ParetoVoting difference is not statistically distinguishable (p=1.000; CI crosses zero), consistent with the negligible Cliff’s Delta (δ=−0.12). Cliff’s Delta effect size analysis, using ParetoVoting as the reference model, reveals large effect sizes for Stacking, LightGBM, Random Forest, SVM, and Logistic Regression (|δ|≥0.52), a moderate effect size for CatBoost (δ=−0.36), and small effect sizes for Optimal Voting (δ=−0.20) and XGBoost (δ=−0.28).

It should be noted that both the Friedman test and the Cliff’s Delta effect sizes were computed over only five paired observations per model (one per outer cross-validation fold, $n_{\mathrm{blocks}}=5$), which constitutes a low-power design for comparing ten models; the reported statistics and effect sizes should therefore be interpreted as indicative of relative model ranking rather than as precise, well-powered hypothesis tests, and this limitation is acknowledged in Section 4. To contextualize these results against uninformative baselines, a majority-class classifier (always predicting the TN class) and a stratified random classifier were evaluated under the identical 5-fold cross-validation protocol. The majority-class baseline achieved F1-macro =0.214±0.001 (Accuracy =0.473, MCC =0.000, by construction, since only one class is ever predicted), while the stratified random baseline achieved F1-macro =0.316±0.016 (Accuracy =0.349, MCC =−0.026, AUROC =0.487±0.010, consistent with chance-level discrimination). All ten evaluated models, including the weakest individual learner (Logistic Regression, F1-macro = 0.642), substantially outperformed both trivial baselines by a wide margin (ΔF1-macro ≥ 0.326 over the stratified random baseline), confirming that the observed classification performance reflects genuine biological signal rather than class-imbalance artifacts or chance agreement.

#### Leave-One-Cohort-Out (LOCO) Validation

To assess cross-cohort generalization within the internal GEO collection, each cohort was held out in turn and the SoftVoting ensemble (fixed hyperparameters, Table 4; fixed 500-gene set, Section 2.5) was trained on the remaining two. Mean macro-F1 across the three held-out cohorts (0.541 ± 0.084; Table 8) was substantially lower than the pooled 5-fold CV estimate (0.735; Table 5), revealing a real cross-cohort generalization gap. Performance was lowest for GSE72094 (F1-macro = 0.444), the largest and most KRAS-enriched cohort (Table 2), with KRAS F1 dropping to 0.265.

### 3.3. TCGA-LUAD Independent External Validation

Generalizability from microarray-based internal cohorts (n=774) to RNA-seq-based TCGA-LUAD (n=508) was tested via independent external validation. After applying ComBat harmonization and class-based probability threshold optimization, the SoftVoting ensemble achieved the following values in external validation: Accuracy = 0.7244, F1-macro = 0.6997, MCC = 0.5427, and AUROC = 0.8504. Class-based results are detailed in Table 9. Bootstrap resampling (1000 iterations) of the TCGA-LUAD cohort yielded 95% confidence intervals of [0.650, 0.745] for F1-macro and [0.816, 0.878] for AUROC (Table 9), confirming the external validation estimate is reasonably precise despite the moderate cohort size.

The calibration split was repeated five times independently (Table 10). EGFR and TN thresholds stayed within their five-split ranges, but the originally reported KRAS threshold (0.15) fell outside its range (0.33–0.50).

External F1-macro across the five sets ranged from 0.664 to 0.707 (argmax baseline: 0.673; originally reported: 0.700). As 0.700 corresponds to the outside-range KRAS threshold, a value near 0.66–0.68 is more representative of expected performance; see Section 4 for discussion. The critical importance of cross-platform harmonization is evident in Table 11. Without the ComBat correction, the model degenerates to predicting only the majority (TN) class for every sample (F1-macro = 0.245, MCC = 0.000)—a degenerate, single-class classifier rather than a genuine competing model. ComBat harmonization alone restores performance to F1-macro = 0.673; subsequent threshold optimization further increases this to F1-macro = 0.700. Because the unharmonized baseline is degenerate, expressing this improvement as a simple fold-change (e.g., “2.9-fold”) would overstate its practical meaning; in absolute terms, ComBat harmonization and threshold calibration together raise F1-macro by 0.455 points (from 0.245 to 0.700), restoring the model from a non-functional, single-class predictor to one with genuine three-class discriminative performance.

**Figure 4:**
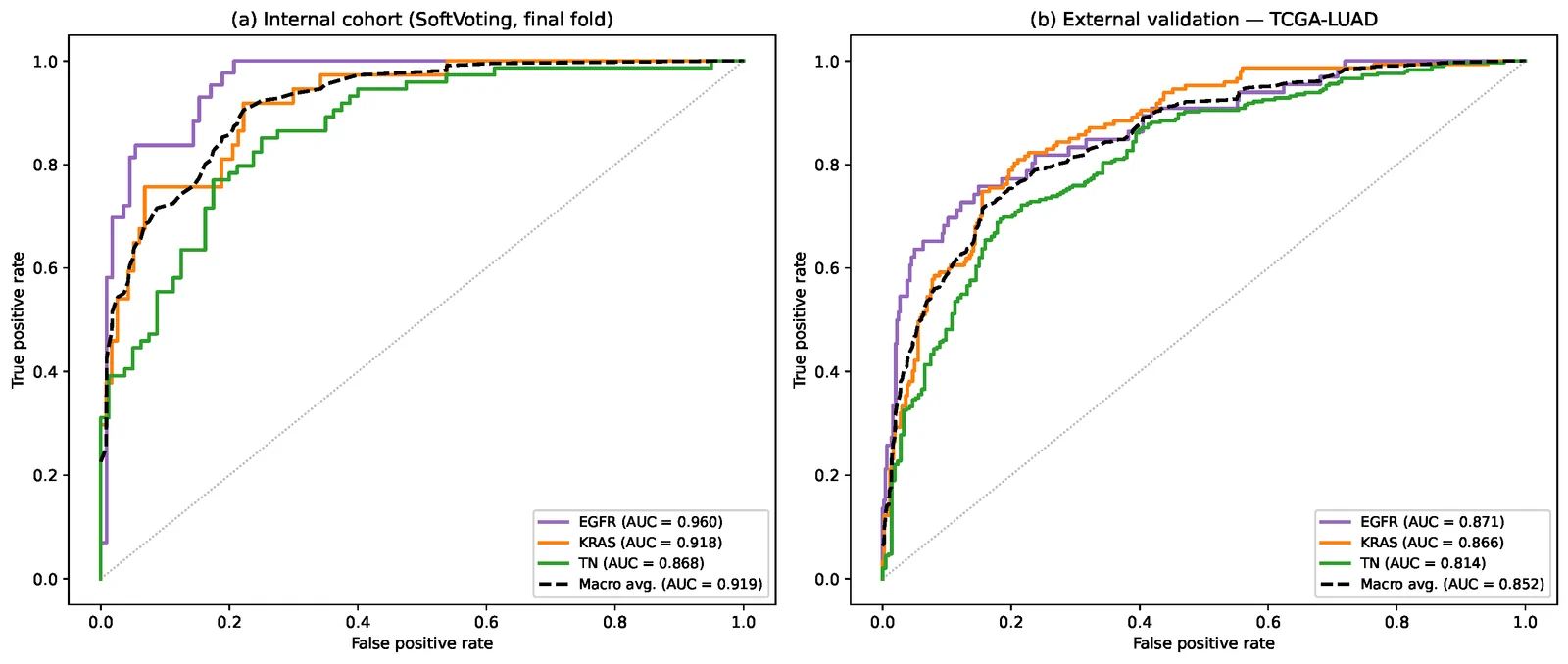
ROC curves for the SoftVoting ensemble. (**a**) Internal cohort—5-fold test set (n=154). (**b**) TCGA-LUAD independent external validation (n=508). A “one-versus-all” (OvR) strategy was applied for each class. The dashed black line indicates the macro-averaged ROC curve, computed by averaging the true-positive rate of each class over a common false-positive-rate grid. This curve-based macro AUC (0.852) differs marginally from the direct macro-average of per-class AUROC values reported elsewhere (0.850, Table 9) due to the different averaging method; both are valid but distinct computations.

ROC curve analysis (Figure 4) demonstrates that the EGFR subtype exhibits the highest discriminatory power in both the internal cohort (AUC = 0.960) and the TCGA external cohort (AUC = 0.871). KRAS demonstrated strong performance on both platforms (internal: AUC = 0.918; TCGA: AUC = 0.866). The relatively low AUC value for the TN subtype in TCGA (0.814) stems from systematic expression differences between the RNA-seq and microarray platforms.

**Figure 5:**
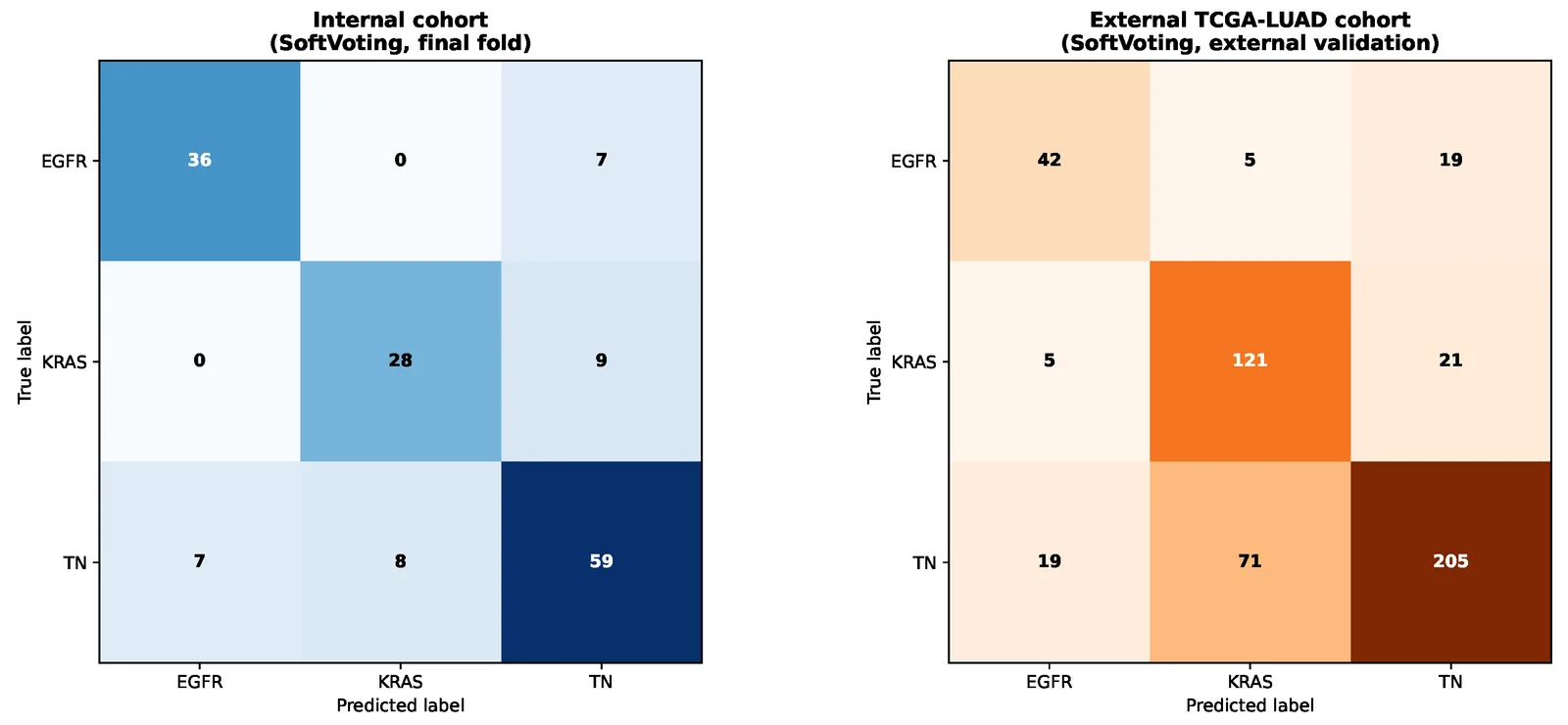
Confusion matrices for the SoftVoting ensemble. (**left**): internal cohort final fold (n=154). (**right**): TCGA-LUAD external validation cohort (n=508). Values indicate sample counts. The predominant misclassification pattern in the TCGA cohort was TN → KRAS (n=71), followed by KRAS → TN (n=21) and EGFR → TN (n=19).

Confusion matrix analysis (Figure 5) identified TN → KRAS misclassification (n=71) as the primary error pattern in the TCGA cohort, followed by KRAS → TN (n=21) in the TCGA cohort; this finding indicates transcriptomic overlap between the KRAS and TN classes under cross-platform conditions.

### 3.4. Calibration and Clinical Utility

To assess whether predicted probabilities are clinically interpretable and whether model-guided decisions offer net clinical value beyond uninformative default strategies, calibration and decision-curve analysis (DCA) were performed on the TCGA-LUAD external validation cohort using a one-vs-rest approach for each class. Calibration was assessed via reliability diagrams and Brier scores (EGFR: 0.073; KRAS: 0.131; TN: 0.175; Figure S4), with all three scores well below the 0.25 threshold expected under chance-level calibration, indicating that predicted probabilities are reasonably well calibrated, with EGFR showing the strongest calibration and TN the weakest, consistent with TN’s comparatively lower discriminative performance reported elsewhere (Table 9). Decision-curve analysis compared the model’s net benefit against “treat-all” and “treat-none” reference strategies across clinically plausible threshold probabilities (1–50%; Figure S5). The model provided greater net benefit than treating all patients across nearly the entire evaluated range for EGFR (threshold range [0.01,0.50]) and KRAS ([0.01,0.50]), and across most of the range for TN ([0.04,0.50]), indicating that model-guided decision-making would be expected to offer clinical benefit over a non-selective treatment strategy across a broad range of plausible decision thresholds.

### 3.5. Ablation Study

The marginal contribution of each pipeline component was systematically quantified through a seven-configuration ablation analysis conducted using 5-fold stratified cross-validation on the internal cohort (n=774), using SoftVoting—the ensemble selected for external validation (Section 2.6.5)—as the reference configuration (Table 12; Figure S3).

The largest performance decreases were observed upon removal of SMOTE oversampling (ΔF1 =−0.022) and SHAP-based feature selection (ΔF1 =−0.018), highlighting the critical importance of class balancing and dimensionality reduction in this imbalanced, high-dimensional setting.

Removal of Bayesian hyperparameter optimization produced a comparable reduction (ΔF1 =−0.018), underscoring the value of the two-stage optimization strategy. Replacing the full heterogeneous ensemble with a single XGBoost classifier resulted in a decline of ΔF1 =−0.012, demonstrating that ensemble diversity contributes complementary discriminative capacity beyond any individual learner. Specifically, excluding the SVM component from the ensemble reduced performance by ΔF1 =−0.013, causing convergence toward the single XGBoost baseline (F1 =0.723), which confirms that the kernel-based SVM generates decision boundaries that are geometrically complementary to gradient boosting models.

Removing ComBat batch correction had a relatively limited effect within the internal cohort (ΔF1 =−0.005), likely because samples within each fold originated from overlapping microarray platforms sharing similar expression distributions. However, as demonstrated in Table 11, the platform-independent contribution of ComBat becomes critically evident during external validation: without harmonization, the model fails to generalize to the RNA-seq TCGA-LUAD cohort (F1-macro =0.245, MCC =0.000), whereas ComBat harmonization restores cross-platform performance to F1-macro = 0.673 prior to threshold calibration. This finding underscores that the internal ablation ΔF1 of ComBat underestimates its true contribution to cross-platform generalizability, which constitutes the central objective of the proposed framework.

A complementary incremental (build-up) ablation, in which pipeline components were added one at a time starting from a single default-parameter XGBoost model on the raw variance-filtered genes, is reported in Table 13. In this build-up order, the largest single gain arose from Bayesian hyperparameter optimization (ΔF1 =+0.047), followed by SHAP-based feature selection (+0.012) and heterogeneous ensembling (+0.012), whereas ComBat (−0.003) and SMOTE (+0.0002) produced negligible changes in macro-F1 within the internal cohort; SMOTE nonetheless increased KRAS F1 from 0.620 to 0.645.

### 3.6. Explanatory Analysis

**Figure 6:**
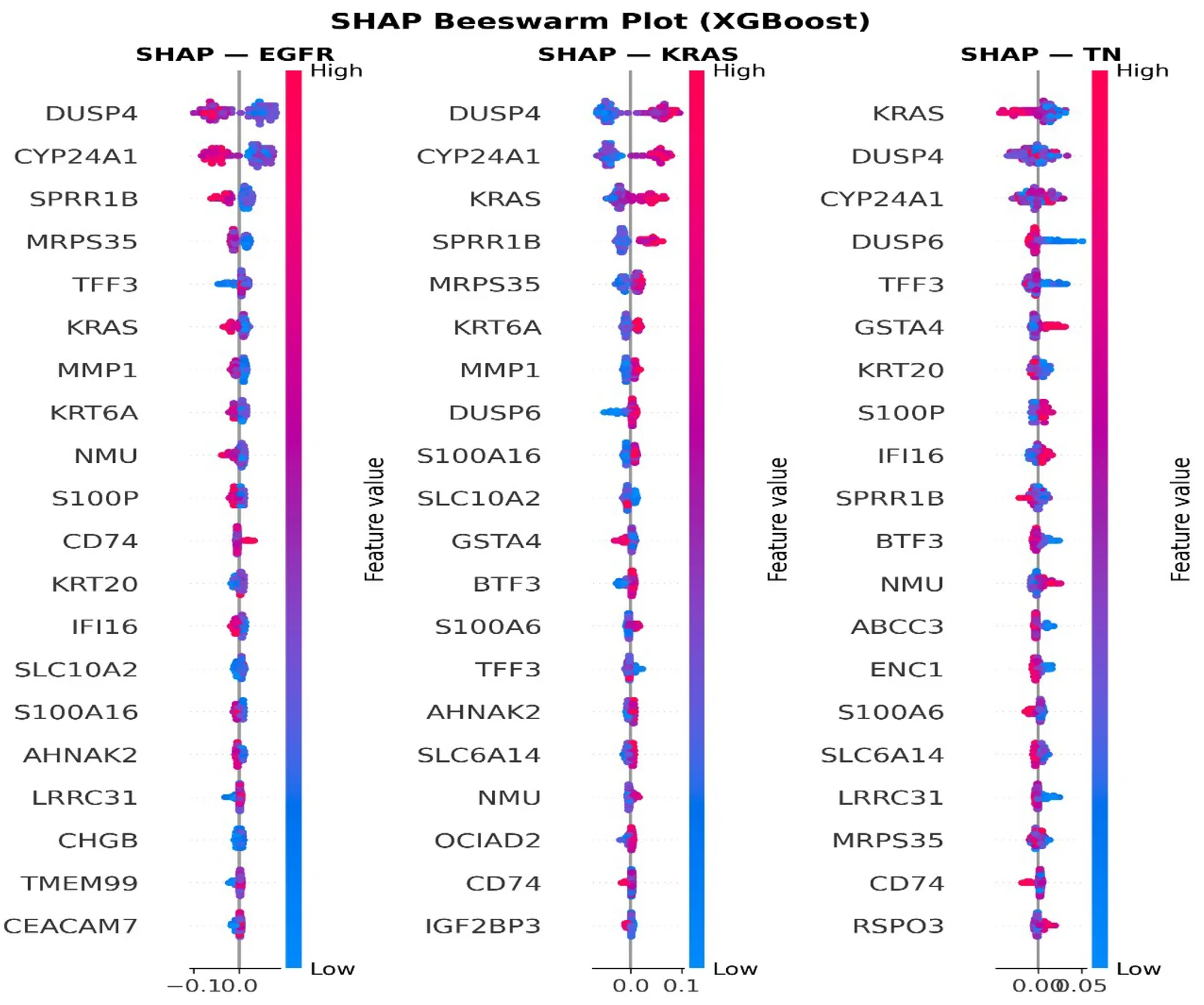
Class-based SHAP beeswarm plots for the XGBoost model (top 20 genes). Each point represents a test sample; the x-axis shows SHAP values, and color indicates feature expression levels (red: high expression, blue: low expression).

Explainability analysis was performed on XGBoost, the primary gradient boosting component of the SoftVoting ensemble. The SHAP Beeswarm analysis illustrates class-based feature contributions in terms of both direction and magnitude. As shown in Figure 6, high expression of DUSP4 and CYP24A1 in the EGFR class directs the model toward an EGFR decision; in the KRAS class, the KRAS gene itself provides the third most significant contribution. These findings are consistent with the literature regarding the expanded SHAP gene set. It has been demonstrated that SPRR1B supports tumor proliferation and metastasis by activating the MAPK pathway in LUAD [38]. MMP1 is a collagenase that activates the KRAS signaling pathway and is associated with tumor invasion in NSCLC; its overexpression has been independently associated with poor prognosis [39]. The dual SHAP contribution of KRAS across both KRAS and TN classes is consistent with previously reported transcriptomic overlap between these subtypes [40]. The fact that low KRAS expression in the TN class exhibits the strongest positive contribution is biologically consistent with the KRAS-negative transcriptomic profile of the TN subtype. The prominence of DUSP4 and CYP24A1 across all three classes suggests that these genes function as shared biological markers in distinguishing the LUAD subtype. DUSP4 is significantly more highly expressed in EGFR-mutant NSCLC cell lines compared to KRAS-mutant cells and plays a critical role in MAPK feedback regulation in EGFR-mutant cells [41]. CYP24A1, on the other hand, has been identified as an independent biomarker associated with poor prognosis, being overexpressed 8–50-fold in LUAD tissue [42]. Completing the global SHAP analysis, LIME local annotation plots reveal individual prediction rationale for representative samples from each class in a model-agnostic manner (Figure S1) [37].

These explainability findings support the biological interpretability of model decisions, aligning with the transparency requirements expected in clinical genomic applications [33]. To assess the biological relevance of the SHAP-selected gene set, pathway enrichment analysis was performed on the top 100 SHAP-ranked genes using the Enrichr API [43]. Reactome pathway analysis revealed significant enrichment in RAF-independent MAPK1/3 activation (FDR =0.020), consistent with the central role of RAS–RAF–MAPK signalling in EGFR- and KRAS-driven LUAD. Additional enriched pathways included Cell Junction Organization (FDR =0.014) and Laminin Interactions (FDR =0.020), reflecting the epithelial remodelling processes characteristic of lung adenocarcinoma. Gene Ontology analysis further identified enrichment in Epithelial Structure Maintenance (FDR =0.032) and Spindle Assembly Checkpoint Signalling (FDR =0.032), consistent with the proliferative and structural dysregulation underlying LUAD subtypes. These findings provide biological validation of the SHAP-selected feature space and support the interpretability of the proposed classification framework. Repeating this enrichment separately per class clarified the origin of the aggregate pathways above: Cell Junction Organization and Laminin Interactions were strongly KRAS-specific (FDR $=2.1\times 10^{-4}$ and $9.6\times 10^{-4}$), while RAF-independent MAPK1/3 Activation was TN-specific (FDR =0.0085). KRAS additionally showed significant ERK/RAS-MAPK feedback enrichment (“ERKs Are Inactivated”, FDR =0.0093), consistent with its established role in RAS/MAPK signaling and ECM remodeling. EGFR showed the same pathways directionally but did not reach FDR significance in this smaller, class-restricted gene set, likely reflecting reduced statistical power. These results extend the KRAS-TN transcriptomic overlap discussed above (Section 4) to shared RAS/MAPK pathway activity, supporting this overlap as genuine biology rather than a modeling artifact.

## 4. Discussion

The drop in the F1-macro value (0.735) obtained in the 5-fold cross-validation of the internal cohort to 0.700 in the TCGA-LUAD external validation can be explained by two main mechanisms. The first is technical platform shift. The model was trained on microarray platforms but tested on RNA-seq data. Differences in dynamic ranges between the two technologies systematically alter normalized expression distributions; while ComBat harmonization mitigates most of this shift—the MCC value drops to 0.000 without harmonization—platform variation continues to limit performance. Beyond cross-platform generalization (tested via TCGA-LUAD), we also assessed cross-cohort generalization within the microarray-based internal data using a Leave-One-Cohort-Out (LOCO) design, re-deriving SHAP feature selection and all hyperparameters independently for each held-out cohort (Table S1). Mean macro-F1 was 0.483±0.077 (range: 0.416–0.566), substantially lower than the pooled 5-fold estimate (F1-macro =0.735), indicating that part of the pooled estimate reflects within-cohort regularities that do not fully transfer across the three GEO cohorts. The second is the biological transcriptomic overlap shared by KRAS and TN subtypes. In the TCGA cohort, the predominant misclassification pattern was TN → KRAS (n=71), followed by KRAS → TN (n=21) and EGFR → TN (n=19). The TN → KRAS pattern suggests that a subset of triple-negative tumors harbours RAS pathway activity at the transcriptomic level, consistent with previously reported overlap between these subtypes [40]. RAS pathway activation is not exclusive to KRAS-mutant tumors; high RAS activity is also detected in 65% of KRAS wild-type LUAD tumors, and these tumors exhibit downstream transcriptomic changes overlapping with those in KRAS-mutant cases [40]. In RTK-RAS-mutated lung adenocarcinoma, it has been demonstrated that the excessive activation of ERK through multiple mechanisms is toxic to tumor cells; this finding suggests that the RAS/ERK signaling level may shape the transcriptomic subtype independently of the presence of a KRAS mutation [44]. This difficulty in transcriptomically separating the three mutational groups is consistent with Planck et al. [15], who, using an independent 96-gene differential-expression signature and a partially overlapping set of microarray cohorts (including GSE31210 and GSE13213, also used in the present study), reported that supervised and unsupervised analyses together were unable to fully resolve EGFR-mutated, KRAS-mutated, and EGFR/KRAS-wild-type adenocarcinomas into distinct transcriptional entities. The present study extends this qualitative, single-platform observation into an explicit, cross-platform (RNA-seq) confusion-matrix characterization, and links the KRAS-TN boundary specifically to a permissive class-decision threshold of 0.15 for KRAS (Section 2.6.6). The instability of the KRAS decision threshold across five independent calibration splits (Table 10; range: 0.33–0.50, mean =0.41±0.06; the originally reported value of 0.15 falls outside this range) is consistent with known small-sample instability in data-driven cutoff selection [45], and with the modest calibration-set size used here (n≈155).

EGFR and TN thresholds were comparatively more stable, suggesting KRAS calibration is disproportionately sensitive to the calibration sample, plausibly reflecting the KRAS–TN transcriptomic overlap discussed above. Across the five repeats, external F1-macro ranged from 0.664 to 0.707 (argmax baseline: 0.673; originally reported value: 0.700), indicating that the thresholds in Table 9 should be interpreted as one plausible calibration rather than a precisely fixed cutoff.

It must be acknowledged that this transcriptomic overlap constitutes a genuine limitation of the proposed framework. The bidirectional KRAS ↔ TN misclassification (KRAS → TN, n=21; TN → KRAS, n=71; Figure 5) implies that the model may produce incorrect treatment guidance for a subset of patients who could benefit from KRAS G12C inhibitors such as sotorasib or adagrasib. A related limitation is that TN was defined solely via EGFR/KRAS mutual exclusivity, without systematic screening for other actionable alterations (ALK, ROS1, BRAF, MET, HER2, RET); the TN group may therefore include a subset of patients with alternative targetable drivers. Similarly, the KRAS-mutant class was defined by mutation presence alone, without distinguishing codon-specific variants (e.g., G12C, G12D, G12V); since only G12C is currently targetable with approved KRAS inhibitors, a KRAS-mutant prediction does not by itself indicate eligibility for this specific therapy. More broadly, the present framework’s clinical premise—that EGFR and KRAS status alone guides first-line treatment selection—reflects the two most common and best-characterized NSCLC drivers, but does not capture the full spectrum of actionable alterations noted above that inform real-world treatment decisions; extending the classification scheme to additional driver genes is an important direction for future work.

Future work should explore the integration of complementary data modalities such as somatic mutation profiles or tumor microenvironment deconvolution signatures to resolve this transcriptomic ambiguity at the classification level. STK11/LKB1 co-mutations, common in KRAS-mutant NSCLC, are also linked to elevated tumor mutational burden and immune-cold phenotypes [3]. TMB was not assessed here, as mutation calls were used only for class labeling; incorporating TMB is a natural extension for future work.

To assess whether immune cell composition contributes to the model’s signal rather than tumor-intrinsic biology, the SHAP-selected 500-gene panel was tested for enrichment against the CIBERSORT LM22 immune signature matrix (547 genes, 22 subsets) [46]. The overlap (18/500 genes) did not exceed chance expectation (hypergeometric test, p=0.43), suggesting the model’s signal predominantly reflects tumor-intrinsic transcriptional programs rather than immune cell infiltration differences; broader deconvolution (e.g., CIBERSORTx, xCell) remains a direction for future work. From a clinical perspective, in cases where tissue biopsy provides insufficient tumor material or cannot be performed due to procedural risks—standard biopsy samples are insufficient for molecular testing in approximately 4–10% of NSCLC patients [7,8,9]—the current pipeline holds potential as a complementary transcriptomic classification tool. This approach aims not to replace standard NGS testing but to provide decision support in cases where testing is not feasible. The fact that the RAF-MEK-ERK signaling cascade produces similar gene expression patterns in both groups inherently complicates expression-based classification [47]. In particular, phenotypic plasticity increases in KRAS-mutant cases accompanied by STK11/LKB1 co-mutations, and the tumor may acquire transcriptomic features specific to the TN group [48].

A further clinical limitation concerns EGFR subtype detection specifically. In the external validation cohort, EGFR sensitivity (recall) was only 0.64 (Table 9), meaning that approximately 36% of true EGFR-mutant patients in the TCGA-LUAD cohort would be missed by the model in its current configuration. This EGFR-recall limitation is further reflected in the error structure itself: EGFR→TN is the third-largest misclassification pattern in the external cohort (n=19; Figure 5), directly contributing to the reduced EGFR sensitivity discussed above. This limitation carries direct clinical weight: EGFR-mutant NSCLC is the subtype with the most established first-line targeted therapy option (TKIs such as erlotinib, gefitinib, and osimertinib), and a false-negative EGFR call could result in an eligible patient being directed toward chemotherapy or immunotherapy pathways rather than TKI-based treatment, with a corresponding delay in receiving the most effective available therapy. This sensitivity limitation is more clinically consequential than aggregate performance metrics such as macro-F1 or accuracy convey, and it reinforces that the current pipeline should be positioned as a complementary triage or prioritization tool for confirmatory molecular testing, rather than a standalone diagnostic replacement, particularly for EGFR subtype calls.

The obtained F1-macro values (internal cohort: 0.735; TCGA: 0.700) should not be directly compared with binary classification studies. Multi-class imbalanced learning remains a structurally more challenging problem compared to binary classification [49,50]. This situation has been clearly documented in the context of cancer genomic classification: While [51] reported that the median accuracy remained at only 44% in a multi-class task covering 21 cancer types, values exceeding 99% can be achieved in the binary cancer-normal classification. The combination of high dimensionality, small sample size, and multi-class imbalance systematically limits the performance of all classifiers [34,52] and methodological standards in this field have not yet fully matured [53]. A further methodological limitation concerns statistical power: the Friedman test and Cliff’s Delta effect sizes reported in Section 3.2 were computed over only five paired fold-level observations per model, which is a low-power design for a ten-model comparison; these results should be interpreted as indicative of relative ranking among models rather than as precisely powered statistical tests, and larger-scale repeated cross-validation would be needed to substantiate the pairwise comparisons with greater statistical confidence. This study provides both a methodological framework for the three-class EGFR/KRAS/TN classification and establishes reference values for future studies.

A related source of optimism in the internal 5-fold CV estimate concerns pooling the three GEO cohorts prior to cross-validation. A Leave-One-Cohort-Out (LOCO) analysis (Section 3.2, Table 8), training on two cohorts and testing on the third in turn, yielded a lower mean macro-F1 (0.541±0.084) than the pooled 5-fold CV estimate (SoftVoting, F1-macro = 0.735). This gap is consistent with prior evidence that ComBat-style batch correction does not fully resolve confounding between batch and outcome so that performance or significance estimates computed after correction remain unreliable relative to genuinely independent evaluation, whether the downstream analysis is classification cross-validation [54] or differential-expression significance testing [55]. Leave-one-dataset-out schemes analogous to the one used here have therefore been increasingly adopted in multi-cohort transcriptomic classification studies for this reason [56]. We report both estimates transparently and note this generalization gap as a limitation for future, larger, batch-aware studies.

This bias is smaller than the worst-case selection-bias scenarios described by Ambroise and McLachlan [57] for non-nested feature selection, but non-negligible: a leak-free check on one held-out fold (Table 3, Section 2.5) found ΔF1-macro of −0.051 (SHAP and hyperparameters re-derived within-fold) narrowing to −0.019 once ComBat was also re-derived the same way. This affects only the internal estimate; the external TCGA-LUAD cohort was never used in feature selection, tuning, or batch correction, and its validation remains unbiased.

## 5. Conclusions

This study presents a reproducible, cross-platform generalizable pipeline for the gene expression-based three-class classification of LUAD driver mutation subtypes (EGFR, KRAS, TN). The integration of NSGA-II Pareto-optimal ensemble weight optimization alongside Bayesian-optimized heterogeneous ensemble learning yielded a ParetoVoting ensemble achieving F1-macro =0.736 in internal 5-fold cross-validation, marginally outperforming the SoftVoting ensemble (F1-macro =0.735; Cliff’s δ=−0.12, negligible). Given this near-equivalence, the simpler SoftVoting ensemble was selected for independent external validation. Reference-batch ComBat harmonization and internal-validation-based probability threshold calibration enabled cross-platform generalization of the SoftVoting ensemble to the RNA-seq-based TCGA-LUAD cohort (F1-macro =0.700, AUROC =0.850); without harmonization, the macro F1 score drops to 0.245. Systematic ablation analysis quantified the marginal contribution of each pipeline component, revealing that not all components contribute equally within internal cross-validation, with ComBat’s critical role instead emerging in cross-platform external generalization. Considering that three-class transcriptomic driver mutation classification lacks comparative reference values in the literature, the obtained performance metrics serve as benchmarks for future studies. Future work should address KRAS–TN transcriptomic ambiguity through single-cell RNA sequencing or complementary mutation data integration, and should evaluate the external generalizability of the Pareto-optimized ensemble weights.

## Figures and Tables

**Table 1:** Comparative literature summary for NSCLC mutation subtype classification.

	Planck et al. (2013) [15]	Li (2022) [14]	Khadirnaikar (2023) [21]	Puiu et al. (2024) [12]	Song (2025) [5]	Almufareh (2026) [6]	Our Study
Input data	Gene expression (microarray)	RNA-seq immune signature	4 omics layers	Binary mutation	Mutation + PPI network	DNA methylation	Gene expression (microarray)
Target	EGFR/KRAS/wt-wt subtype	NSCLC clusters	NSCLC clusters	LUAD/LUSC histology	Cancer subtype	LUAD/LUSC histology	EGFR/KRAS/TN mutation subtype
Data source	9 microarray cohorts (GEO)	TCGA	TCGA	TCGA single platform	TCGA	TCGA + GEO	GEO (3 cohorts) + TCGA
Batch correction	None (per-cohort analysis)	None	None	None	None	None	ComBat
Imbalance handling	None	None	None	Weighted sampler	Unspecified	None	SMOTE
Model	ANOVA differential expression + hierarchical clustering (96-gene sig.)	RF/SVM/XGB/ AdaBoost	AE + K-means + fusion	MLP + regularization	OAO-SVM	Deep neural network	Ensemble (XGB+LGBM+RF +CatBoost+SVM)
Optimization	None	Grid search	None	Adam (fixed LR)	Grid search	None	Bayesian optimization (Optuna)
Interpretability	Differential expression signature	None	None	Error analysis	None	CpG biomarker	Feature selection + biomarker discovery
External validation	4 microarray cohorts (same-platform)	None	None	None	None	GEO	TCGA-LUAD (n=508) (cross-platform, RNA-seq)
Mutational subtype	EGFR/KRAS/wt-wt	None	None	None	None	None	EGFR/KRAS/TN
Multiple cohort harmonization	None (analyzed individually)	None	None	None	None	None	3 GEO cohorts

**Table 2:** Datasets used for model development and external validation. TN: triple-negative. TCGA-LUAD expression profiles were obtained from UCSC Xena; mutation labels were derived from the MC3 somatic mutation dataset.

Dataset	Platform	*n* ^a^	EGFR	KRAS	TN	Role
GSE31210	Affymetrix GPL570	226 ^b^	127	20	68	Internal Cohort
GSE13213	Agilent GPL6480	117	45	15	57	Internal Cohort
GSE72094	Affymetrix GPL15048	442	47	154	241	Internal Cohort
TCGA-LUAD	RNA-seq (Xena HiSeqV2)	508	66	147	295	External Validation

^a^ The *n* column reports the total number of samples originally available in each cohort. The EGFR, KRAS, and TN columns report sample counts after exclusion of ALK fusion-positive cases (see Section 2.1). ^b^ GSE31210’s n=226 includes 11 ALK fusion-positive samples that were excluded prior to model training and evaluation (127+20+68=215 after exclusion); GSE13213 and GSE72094 were not screened for ALK status in the present analysis (see Section 2.1); their *n* values equal the sum of the EGFR, KRAS, and TN columns.

**Table 3:** Leak-free sensitivity check for one held-out outer fold (fold 2/5). Each row re-derives the listed components using only that fold’s training partition; all other pipeline steps are unchanged.

Re-Derived Components	Fixed Pipeline F1	Leak-Free F1	ΔF1
SHAP + hyperparameters	0.726	0.676	−0.051
ComBat + SHAP + hyperparameters	0.726	0.707	−0.019

**Table 4:** Optimized hyperparameters for all base classifiers. Bayesian optimization was performed with 50 trials per model using 3-fold cross-validation and macro F1 as the objective. The TPE sampler (Optuna v3.x) was used with a fixed random seed (42).

Model	Method	Optimized Hyperparameters
XGBoost	Bayesian (TPE)	n_estimators = 553, max_depth = 9, lr = 0.0212, subsample = 0.503, colsample_bytree = 0.779, min_child_weight = 7, gamma = 1.032, alpha = 0.010, lambda = 0.052
LightGBM	Bayesian (TPE)	n_estimators = 194, max_depth = 11, lr = 0.143, num_leaves = 159, subsample = 0.785, colsample_bytree = 0.463, min_child_samples = 21, alpha = 0.00013, lambda = 0.450
Random Forest	Bayesian (TPE)	n_estimators = 500, max_depth = 13, min_samples_split = 13, min_samples_leaf = 5, max_features = 0.3
CatBoost	Bayesian (TPE)	iterations = 455, depth = 6, lr = 0.137, l2_leaf_reg = 1.648, border_count = 183, bagging_temperature = 0.986
SVM	Bayesian (TPE)	C = 1.541, kernel = rbf, gamma = auto
Logistic Regression	Fixed	C = 0.0104, solver = lbfgs, max_iter = 2000, class_weight = balanced

**Table 5:** Five-fold cross-validation results (n=774, internal cohort; mean ± SD). Simple, interpretable baselines are reported separately below. Bold indicates the best-performing model and its best F1-macro, accuracy, and MCC values.

Model	F1-Macro	±SD	Accuracy	MCC	AUROC	AUROC ± SD
**ParetoVoting**	**0.7364**	0.023	**0.7365**	**0.5826**	0.8772	0.020
SoftVoting	0.7350	0.036	0.7352	0.5805	0.8773	0.019
Optimal Voting	0.7323	0.029	0.7326	0.5763	0.8751	0.021
XGBoost	0.7226	0.038	0.7223	0.5612	0.8747	0.019
Stacking	0.7155	0.037	0.7197	0.5535	0.8699	0.020
LightGBM	0.7104	0.027	0.7107	0.5429	0.8644	0.021
CatBoost	0.7090	0.035	0.7068	0.5392	0.8648	0.024
Random Forest	0.7032	0.028	0.7029	0.5315	0.8639	0.022
SVM	0.6989	0.034	0.7029	0.5277	0.8651	0.019
Logistic Regression	0.6417	0.038	0.6383	0.4440	0.8062	0.028
Elastic-Net LR	0.6295	0.036	0.6267	0.4233	0.7967	0.030
Nearest Centroid	0.5735	0.026	0.5736	0.3729	0.7748	0.024
Linear SVM	0.5825	0.032	0.5840	0.3552	0.7715	0.031
Majority-Class Baseline	0.2140	0.001	0.4729	0.0000	—	—
Stratified Random Baseline	0.3157	0.016	0.3488	−0.0257	0.4872	0.010

**Table 6:** Results of paired statistical comparisons against the reference model, ParetoVoting (Friedman test: $\chi^{2}=23.79$, p=0.0046, df=9). For each model, the Wilcoxon signed-rank *p*-value, the paired 95% CI of the mean fold-level F1-macro difference, and Cliff’s δ are reported relative to ParetoVoting across the five outer folds. Bold denotes the reference model.

Model	F1 (Mean ± SD)	Wilcoxon *p*	95% CI of Paired Diff.	Cliff’s δ	Effect Size
**ParetoVoting**	**0.736 ± 0.023**	—	—	—	—
SoftVoting	0.735 ± 0.036	1.000	[−0.021, 0.018]	−0.12	Negligible
Optimal Voting	0.732 ± 0.029	0.312	[−0.012, 0.004]	−0.20	Small
XGBoost	0.723 ± 0.038	0.438	[−0.045, 0.017]	−0.28	Small
Stacking	0.716 ± 0.037	0.125	[−0.049, 0.007]	−0.52	Large
LightGBM	0.710 ± 0.027	0.125	[−0.052, 0.000]	−0.52	Large
CatBoost	0.709 ± 0.035	0.125	[−0.067, 0.012]	−0.36	Moderate
Random Forest	0.703 ± 0.028	0.062	[−0.047, −0.019]	−0.68	Large
SVM	0.699 ± 0.034	0.062	[−0.069, −0.006]	−0.68	Large
Logistic Regression	0.642 ± 0.038	0.062	[−0.131, −0.058]	−1.00	Large

**Table 7:** Class-specific F1 in the internal cohort (5-fold CV) and TCGA-LUAD external validation. Bold indicates the primary reported model.

Cohort	Model	EGFR F1	KRAS F1	TN F1
Internal Cohort (5-Fold CV)	**ParetoVoting**	**0.756 ± 0.038**	**0.720 ± 0.051**	**0.733 ± 0.025**
	SoftVoting	0.756 ± 0.048	0.717 ± 0.061	0.732 ± 0.033
TCGA-LUAD (External Validation)	SoftVoting + threshold	0.64	0.70	0.76

**Table 8:** Leave-One-Cohort-Out (LOCO) validation results for the SoftVoting ensemble. Each row reports performance on the named cohort when it was excluded from training and the ensemble was trained only on the remaining two GEO cohorts.

Held-Out Cohort	*n*	Accuracy	F1-Macro	MCC	F1 (EGFR)	F1 (KRAS)	F1 (TN)
GSE13213	117	0.598	0.583	0.354	0.471	0.615	0.661
GSE31210	215	0.600	0.595	0.404	0.646	0.583	0.556
GSE72094	442	0.518	0.444	0.232	0.426	0.265	0.641
Mean ± SD	—	0.572±0.044	0.541±0.084	0.330±0.089	—	—	—

**Table 9:** TCGA-LUAD external validation results for the SoftVoting ensemble (n=508). Applied class-specific thresholds were: EGFR = 0.30, KRAS = 0.15, and TN = 0.34. Thresholds were optimized on an internal validation subset, ensuring independence of the external validation cohort. 95% CIs (bootstrap, 1000 resamples of the TCGA cohort) are given in brackets for macro-averaged metrics.

Class	Sensitivity	Precision	F1	AUROC
EGFR (n=66)	0.64	0.64	0.64	0.871
KRAS (n=147)	0.82	0.61	0.70	0.866
TN (n=295)	0.69	0.84	0.76	0.814
Macro Average	0.72	0.70	0.70 [0.650, 0.745]	0.850 [0.816, 0.878]
Overall Accuracy = 0.7244 [0.681, 0.764] MCC = 0.5427 [0.472, 0.609]				

**Table 10:** Per-class decision threshold stability across five independent stratified calibration splits (Section 2.6.6). “Original” denotes the single-split threshold reported in Table 9; “Inside range” indicates whether this value falls within the five-split range. Bold marks the original threshold that falls outside its five-split range.

Class	Min	Max	Mean ± SD	Original	Inside Range
EGFR	0.15	0.48	0.330 ± 0.118	0.30	Yes
KRAS	0.33	0.50	0.406 ± 0.063	0.15	**No**
TN	0.24	0.43	0.350 ± 0.064	0.34	Yes

**Table 11:** Effect of ComBat harmonization and threshold optimization on TCGA-LUAD external validation performance (n=508) using the SoftVoting ensemble. Threshold optimization was performed on an internal validation subset; Change denotes the difference relative to the full configuration.

Configuration	F1-Macro	Accuracy	MCC	AUROC	Change
ComBat + Threshold Optimization	0.6997	0.7244	0.5427	0.8504	Reference
ComBat Enabled, Threshold Optimization Disabled	0.6728	0.7106	0.4816	0.8504	−0.027
No ComBat (No Harmonization)	0.2449	0.5807	0.0000	0.5803	−0.455

**Table 12:** Ablation study results for the SoftVoting ensemble using 5-fold cross-validation on the internal cohort (n=774). ΔF1 denotes the change relative to the full pipeline configuration. The full pipeline served as the reference model.

Configuration	F1-Macro	±SD	Accuracy	MCC	AUROC	ΔF1
Full Pipeline	0.7350	0.036	0.7352	0.5805	0.8773	—
No ComBat	0.7298	0.024	0.7287	0.5727	0.8738	−0.0052
No SMOTE	0.7127	0.029	0.7158	0.5485	0.8776	−0.0223
No SHAP (all 2000 genes)	0.7166	0.030	0.7197	0.5547	0.8677	−0.0184
No Bayesian Optimization	0.7175	0.029	0.7184	0.5530	0.8715	−0.0175
XGBoost only (no ensemble)	0.7226	0.039	0.7223	0.5612	0.8747	−0.0124
Ensemble Excluding SVM	0.7217	0.035	0.7210	0.5591	0.8733	−0.0133

**Table 13:** Incremental (build-up) ablation (n=774, internal cohort, 5-fold CV); each step adds one pipeline component to the previous configuration. Steps 1–5 use a single classifier; Step 6 is the SoftVoting ensemble and Step 7 is ParetoVoting. Class-wise F1 (EGFR/KRAS/TN) is reported for Steps 3–4 to isolate SMOTE’s per-class effect. Bold indicates the best-performing step and its best F1-macro, accuracy, and MCC values.

Configuration	F1-Macro	±SD	Accuracy	MCC	AUROC	ΔF1	Class-Wise F1 (EGFR/KRAS/TN)
Step 1: Baseline (raw genes, single XGB, default HP, no SMOTE)	0.6666	0.039	0.6796	0.4872	0.8364	—	—
Step 2: + ComBat batch correction	0.6635	0.043	0.6719	0.4764	0.8367	−0.0031	—
Step 3: + SHAP feature selection (500 genes)	0.6751	0.038	0.6835	0.4952	0.8481	+0.0116	0.710/0.620/0.695
Step 4: + SMOTE class balancing	0.6753	0.024	0.6758	0.4910	0.8468	+0.0002	0.712/0.645/0.668
Step 5: + Bayesian-optimized hyperparameters (single XGB)	0.7226	0.039	0.7223	0.5612	0.8747	+0.0473	—
Step 6: + Heterogeneous ensemble (SoftVoting)	0.7350	0.036	0.7352	0.5805	0.8773	+0.0124	—
**Step 7: ParetoVoting (NSGA-II weights, same splits/features as Step 6)**	**0.7364**	0.023	**0.7365**	**0.5826**	0.8772	+0.0014	—

## Data Availability

The gene expression datasets analyzed in this study are publicly available. GSE31210, GSE13213, and GSE72094 are accessible via the Gene Expression Omnibus (GEO) repository (https://www.ncbi.nlm.nih.gov/geo/, accessed on 18 October 2026). TCGA-LUAD RNA-seq expression profiles were obtained from the UCSC Xena platform (https://xenabrowser.net/datapages/, accessed on 18 October 2026); somatic mutation labels were derived from the MC3 mutation call file available through the GDC Data Portal (https://portal.gdc.cancer.gov/, accessed on 18 October 2026). The complete analysis pipeline, including ComBat harmonization, SHAP-based feature selection, Bayesian hyperparameter optimization, and heterogeneous ensemble classification, is publicly available at https://github.com/SibelKervanci/nsclc-driver-mutation-classification (accessed on 18 October 2026).

## Supplementary Materials

The following supporting information can be downloaded at: https://www.mdpi.com/article/10.3390/diagnostics16203413/s1, Figure S1: LIME local interpretability analysis—representative examples per class (XGBoost model): EGFR sample (left), KRAS sample (middle), and TN sample (right). Blue bars indicate gene features that increase the probability of the corresponding class (positive LIME contribution), while red bars indicate features that decrease it (negative LIME contribution). Figure S2: Five-fold cross-validation model comparison—macro F1 mean ± standard deviation. Dark red: SoftVoting (fixed-weight ensemble, carried forward for external validation); firebrick: ParetoVoting (NSGA-II-optimized ensemble, statistically equivalent to SoftVoting); light red (salmon): OptimalVoting and Stacking; blue: single classifiers. Figure S3: Ablation study results showing the marginal contribution of pipeline components to macro F1 (5-fold CV, n=774). Green: full pipeline reference; blue: ablation configurations. The dashed green line indicates the full pipeline performance (F1=0.735). Figure S4: Calibration (reliability) diagram for the SoftVoting ensemble on the TCGA-LUAD external validation cohort (n=508), computed one-vs-rest for each class (purple: EGFR; orange: KRAS; green: TN). Each point shows the observed frequency of the class against the mean predicted probability within a probability bin; the dashed grey line indicates perfect calibration. Brier scores: EGFR 0.073, KRAS 0.131, TN 0.175. Figure S5: Decision-curve analysis for the SoftVoting ensemble on the TCGA-LUAD external validation cohort (n=508), computed one-vs-rest for EGFR (left), KRAS (middle), and TN (right). Solid lines show the model’s net benefit across threshold probabilities of 0.01–0.50; the dashed grey line represents the “treat-all” strategy and the dotted black line the “treat-none” strategy (net benefit = 0). Table S1: Leave-One-Cohort-Out (LOCO) cross-cohort validation results. For each held-out cohort (GSE31210, GSE13213, GSE72094), SHAP-based feature selection and all model hyperparameters were independently re-derived using only the remaining two cohorts’ training data.
