# Supplementary material for: Multi-Platform Transcriptomic Classification of LUAD Driver Mutation Subtypes via Heterogeneous Ensemble Learning
Source: Diagnostics (Basel). 2026 Oct 20;16(20):3413. doi: 10.3390/diagnostics16203413 (PMC13703602; doi:10.3390/diagnostics16203413)
Supplement: Supplementary file 1 [file diagnostics-16-03413-s001.zip › diagnostics-4581051-supplementary.pdf]

Supplementary Materials: Multi-Platform Transcriptomic Classification of LUAD Driver Mutation Subtypes via Heterogeneous Ensemble Learning

Ilkay Sibel Kervancı and Gözde Özsert Yiğit *

1. Supplementary Figures

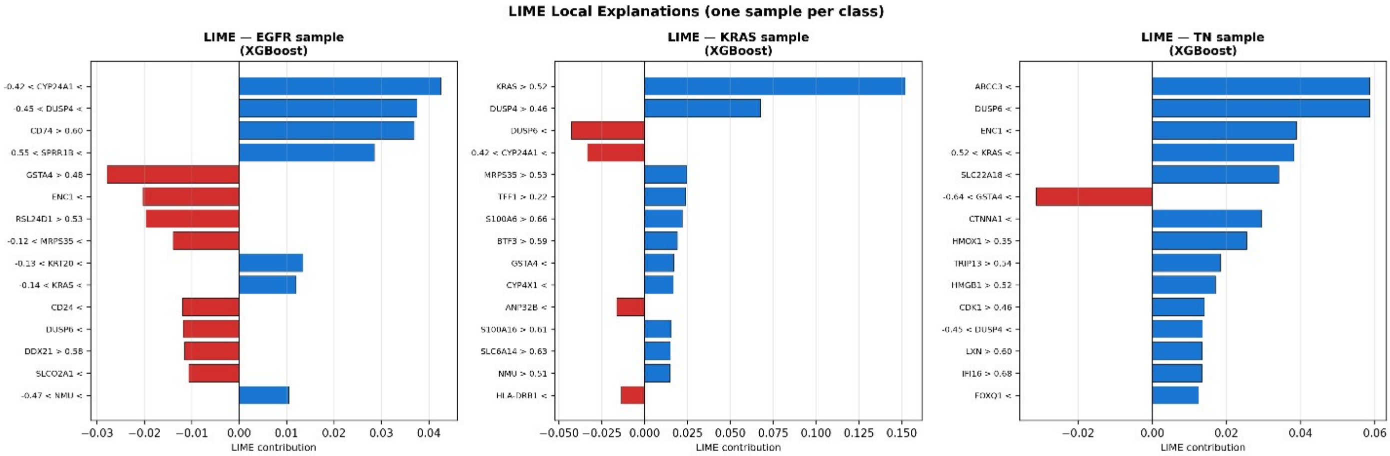

Figure S1. LIME local interpretability analysis—representative examples per class (XGBoost model): EGFR sample (**left**), KRAS sample (**middle**), and TN sample (**right**). Blue bars indicate gene features that increase the probability of the corresponding class (positive LIME contribution), while red bars indicate features that decrease it (negative LIME contribution).

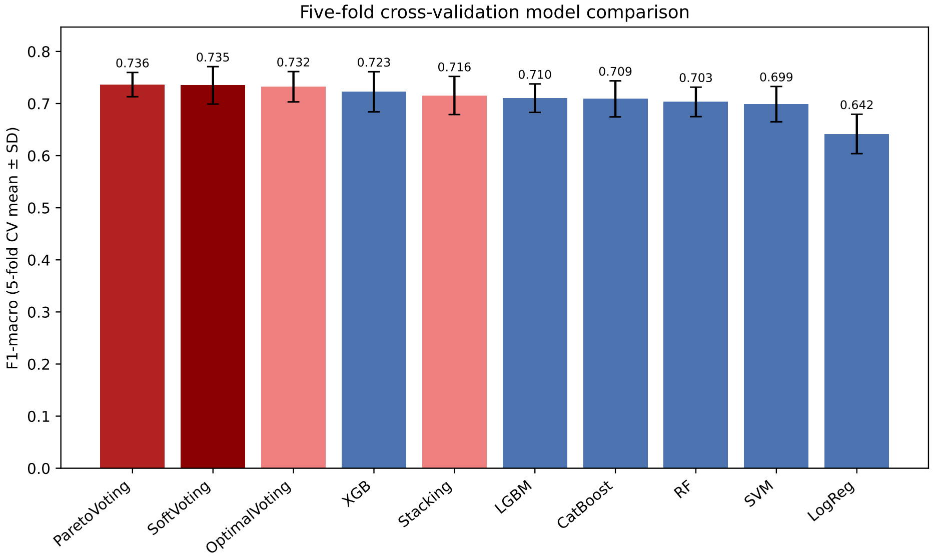

Figure S2. Five-fold cross-validation model comparison—macro F1 mean ± standard deviation ($n = 774$). Dark red: SoftVoting (fixed-weight ensemble, carried forward for external validation); firebrick: ParetoVoting (NSGA-II-optimized ensemble, statistically equivalent to SoftVoting); light red (salmon): OptimalVoting and Stacking; blue: single classifiers.

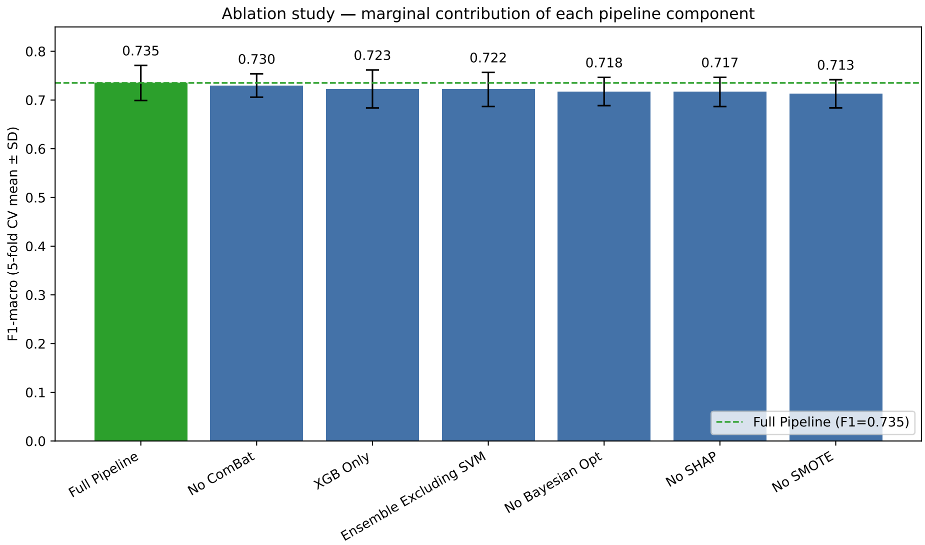

Figure S3. Ablation study results showing the marginal contribution of pipeline components to macro F1 (5-fold CV, $n = 774$). Green: full pipeline reference; blue: ablation configurations. The dashed green line indicates the full pipeline performance (F1 = 0.735).

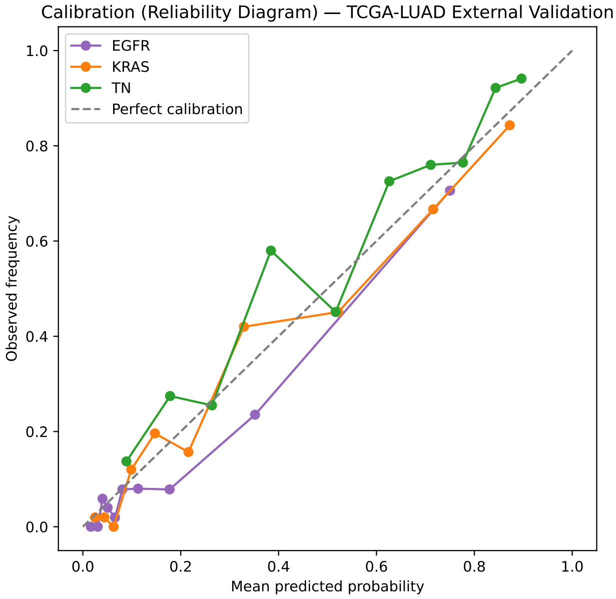

Figure S4. Calibration (reliability) diagram for the SoftVoting ensemble on the TCGA-LUAD external validation cohort ($n = 508$), computed one-vs-rest for each class (purple: EGFR; orange: KRAS; green: TN). Each point shows the observed frequency of the class against the mean predicted probability within a probability bin; the dashed grey line indicates perfect calibration. Brier scores: EGFR 0.073, KRAS 0.131, TN 0.175.

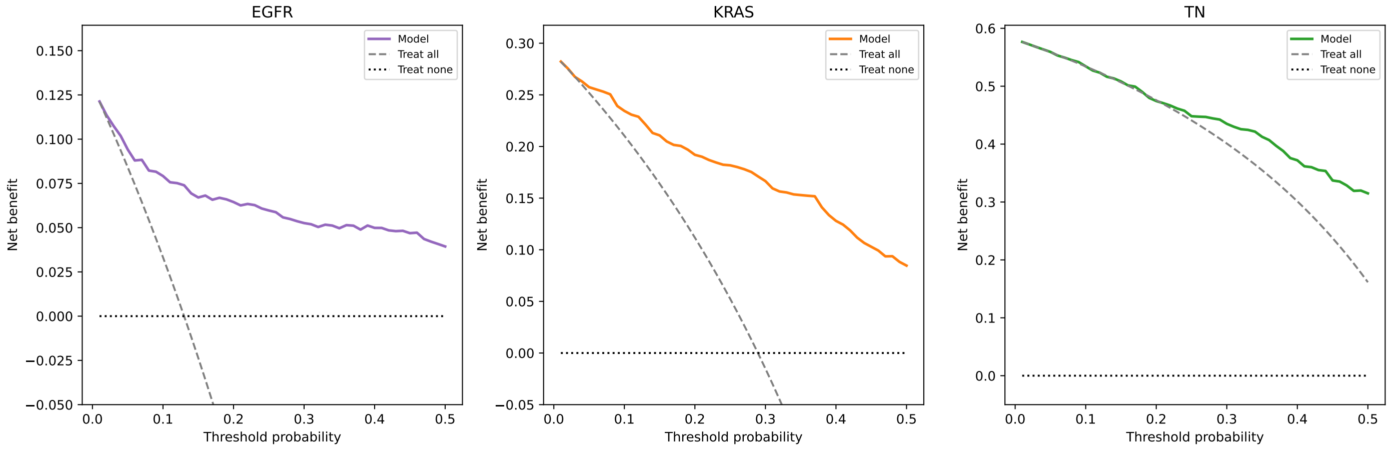

Figure S5. Decision-curve analysis for the SoftVoting ensemble on the TCGA-LUAD external validation cohort ($n = 508$), computed one-vs-rest for EGFR (**left**), KRAS (**middle**), and TN (**right**). Solid lines show the model's net benefit across threshold probabilities of 0.01–0.50; the dashed grey line represents the "treat-all" strategy and the dotted black line the "treat-none" strategy (net benefit = 0).

2. Supplementary Table

Table S1. Leave-One-Cohort-Out (LOCO) cross-cohort validation results. For each held-out cohort (GSE31210, GSE13213, GSE72094), SHAP-based feature selection and all model hyperparameters (XGBoost, LightGBM, Random Forest, CatBoost, SVM) were independently re-derived using only the remaining two cohorts' training data, with no information from the held-out cohort involved in any selection step. ComBat harmonization was applied jointly across all three cohorts, consistent with the main pipeline design.

Held-Out Cohort	n (Test)	F1-Macro	Accuracy	MCC
GSE31210	215	0.4657	0.4465	0.2303
GSE13213	117	0.5663	0.5726	0.3037
GSE72094	442	0.4156	0.4887	0.2027
Mean ± SD	—	0.4825 ± 0.0767	—	—

Note: Mean macro-F1 across the three LOCO iterations (0.4825 ± 0.0767) is substantially lower than the pooled internal 5-fold cross-validation estimate (F1-macro = 0.735), indicating that a component of the pooled estimate reflects within-cohort regularities that do not fully transfer across the three constituent GEO cohorts.
